# NuMA is a mitotic adaptor protein that activates dynein and connects it to microtubule minus ends

**DOI:** 10.1083/jcb.202408118

**Published:** 2025-02-11

**Authors:** Sabina Colombo, Christel Michel, Silvia Speroni, Felix Ruhnow, Maria Gili, Cláudia Brito, Thomas Surrey

**Affiliations:** 1 Centre for Genomic Regulation (CRG), The Barcelona Institute of Science and Technology, Barcelona, Spain; 2 Universitat Pompeu Fabra (UPF), Barcelona, Spain; 3 Catalan Institution for Research and Advanced Studies (ICREA), Barcelona, Spain

## Abstract

Nuclear mitotic apparatus protein (NuMA) is indispensable for the mitotic functions of the major microtubule minus-end directed motor cytoplasmic dynein 1. NuMA and dynein are both essential for correct spindle pole organization. How these proteins cooperate to gather microtubule minus ends at spindle poles remains unclear. Here, we use microscopy-based in vitro reconstitutions to demonstrate that NuMA is a dynein adaptor, activating processive dynein motility together with dynein’s cofactors dynactin and Lissencephaly-1 (Lis1). Additionally, we find that NuMA binds and stabilizes microtubule minus ends, allowing dynein/dynactin/NuMA to transport microtubule minus ends as cargo to other minus ends. We further show that the microtubule-nucleating γ-tubulin ring complex (γTuRC) hinders NuMA binding and that NuMA only caps minus ends of γTuRC-nucleated microtubules after γTuRC release. These results provide new mechanistic insight into how dynein, dynactin, NuMA, and Lis1 together with γTuRC and uncapping proteins cooperate to organize spindle poles in cells.

## Teaser

Using microscopy-based in vitro reconstitutions, Colombo et al. show that NuMA is a mitotic dynein adaptor that binds and caps microtubule minus ends, explaining how dynein, NuMA, dynactin, and Lis1 focus microtubule minus ends at mitotic spindle poles.

## Introduction

Cytoplasmic dynein 1 (henceforth dynein) is the major microtubule minus-end–directed motor protein in animal cells. In interphase, dynein is essential for the retrograde transport of a multitude of cargoes, such as vesicles and organelles (Yildiz and Zhao, 2023). During mitosis, it participates in nuclear envelope breakdown and mitotic spindle organization and function (Raaijmakers and Medema, 2014). Human dynein is a large protein complex (≈1.5 MDa) consisting of six distinct polypeptides, each present in duplicate. The C-terminal portion of the heavy chain forms the motor domain consisting of a ring of six AAA domains connected to a microtubule-binding domain by a stalk (Canty et al., 2021). The N-terminal part together with the smaller subunits forms the tail, which serves as a docking site for regulatory components and dynein’s cargo (Reck-Peterson et al., 2018).

When not bound to microtubules, dynein predominantly exists in an autoinhibited “Phi” conformation (Torisawa et al., 2014; Zhang et al., 2017). The dynein regulator Lis1 can alleviate this autoinhibition, acting as a molecular wedge that separates the two dynein motor domains (Karasmanis et al., 2023), allowing dynein to bind other interaction partners and modulating dynein’s microtubule-binding affinity (Gillies et al., 2022). Dynactin is another large protein complex (≈1.1 MDa) formed by 23 subunits of 11 different polypeptides, including a central actin-like polymer, the Arp1 filament (Urnavicius et al., 2015). Dynactin serves as a cofactor for virtually all known dynein activities (Canty and Yildiz, 2020). For processive motility, dynein and dynactin need to associate with an adaptor (McKenney et al., 2014; Schlager et al., 2014). Dynein adaptors are coiled-coil proteins whose N-terminal part is sandwiched in between dynein and dynactin, thereby stabilizing dynein’s and dynactin’s active conformation (Chowdhury et al., 2015; Chaaban and Carter, 2022). Lis1 promotes the recruitment of two dynein dimers per dynactin and reinforces the tethering to dynactin (Elshenawy et al., 2020; Htet et al., 2020; Singh et al., 2024). The C-terminal part of dynein adaptors contains the cargo-binding domain (Carter et al., 2016; Olenick and Holzbaur, 2019). Therefore, in addition to promoting dynein activation, adaptors bind specific cargoes, providing the dynein/dynactin complex with functional versatility (Olenick and Holzbaur, 2019; Canty and Yildiz, 2020).

During cell division, dynein is indispensable for the correct functioning of meiotic and mitotic spindles. One of its important roles is spindle pole focusing, thought to be achieved by motor-driven gathering of microtubule minus ends (Verde et al., 1991; Heald et al., 1996; Sikirzhytski et al., 2014; Hueschen et al., 2019; So et al., 2022). How dynein crosslinks microtubules and transports minus ends toward other minus ends remains however unclear.

Dynein’s pole-focusing activity requires its ubiquitous partners, dynactin and Lis1 (Wang et al., 2013; Monda and Cheeseman, 2018; So et al., 2022). Moreover, it requires also a mitosis-specific interaction partner, the nuclear mitotic apparatus protein (NuMA). In animal cells, NuMA localizes to the nucleus during interphase. In mitosis, it accumulates at spindle poles to contribute to proper pole organization (Lydersen and Pettijohn, 1980; Maekawa et al., 1991; Gaglio et al., 1995; Merdes et al., 1996, 2000; Heald et al., 1997; Hueschen et al., 2017) and recruits dynein to the cell cortex to ensure correct spindle positioning (Okumura et al., 2018).

NuMA assembles into homodimers via its long central coiled-coil (≈210 nm) (Harborth et al., 1995; Harborth et al., 1999; Forth et al., 2014). Its N-terminal Hook domain binds the dynein light intermediate chain and is adjacent to a CC1-box-like motif conserved among various dynein adaptors (Renna et al., 2020). A Spindly-like motif has also been identified (Okumura et al., 2018; Tsuchiya et al., 2021), which may promote the association with dynactin’s pointed end (Gama et al., 2017; Lee et al., 2020). NuMA co-immunoprecipitates with dynein and dynactin in *Xenopus* egg extract (Merdes et al., 1996), and its first 505 amino acids are sufficient for cortical recruitment of dynein in human cells (Okumura et al., 2018). Although NuMA has therefore been proposed to function as an activating dynein adaptor (Hueschen et al., 2017; Reck-Peterson et al., 2018; Renna et al., 2020), this has not been directly demonstrated yet.

NuMA’s C-terminal part has been shown to be required for correct spindle organization in human cells (Hueschen et al., 2017; Okumura et al., 2018; Pirovano et al., 2019). It interacts with microtubules through two proposed microtubule binding domains (MTBDs) (Du et al., 2002; Gallini et al., 2016; Chang et al., 2017). Moreover, in vitro experiments with purified proteins revealed that the C-terminal part can also support NuMA’s self-assembly into oligomers (Harborth et al., 1999) and trigger phase separation (Sun et al., 2021), which may explain NuMA’s clustering behavior observed in cells (Okumura et al., 2018), and may contribute to passive microtubule crosslinking (Merdes et al., 1996; Nachury et al., 2001; Haren and Merdes, 2002). In human cells, NuMA has also been shown to localize to the minus ends of laser-ablated kinetochore fibers independently of dynein (Hueschen et al., 2017), raising the possibility that NuMA has the intrinsic property of recognizing microtubule minus ends, which has however not been tested directly. The molecular mechanism by which NuMA contributes to dynein’s microtubule minus-end gathering activity remains therefore unclear.

Here, we investigate the interplay between NuMA, dynein, and microtubules using total internal reflection fluorescence (TIRF) microscopy–based in vitro reconstitution assays with purified proteins. We find that the N-terminal part of NuMA can activate processive dynein motility and that this activation does not only require dynactin but also Lis1. We demonstrate that NuMA’s C-terminal part directly binds microtubules with a preference for free minus ends, capping and stabilizing them. Finally, we show that the dynein/dynactin/NuMA complex can transport the minus ends of cargo microtubules toward the minus ends of other microtubules. This establishes NuMA as an activating dynein adaptor, whose cargo is a microtubule minus end. These results provide mechanistic insight into the molecular mechanism by which dynein, dynactin, NuMA, and Lis1 cooperate to focus spindle poles during mitosis.

## Results

### NuMA is a dynein adaptor that requires Lis1 and dynactin to activate dynein motility

**Figure 1.**
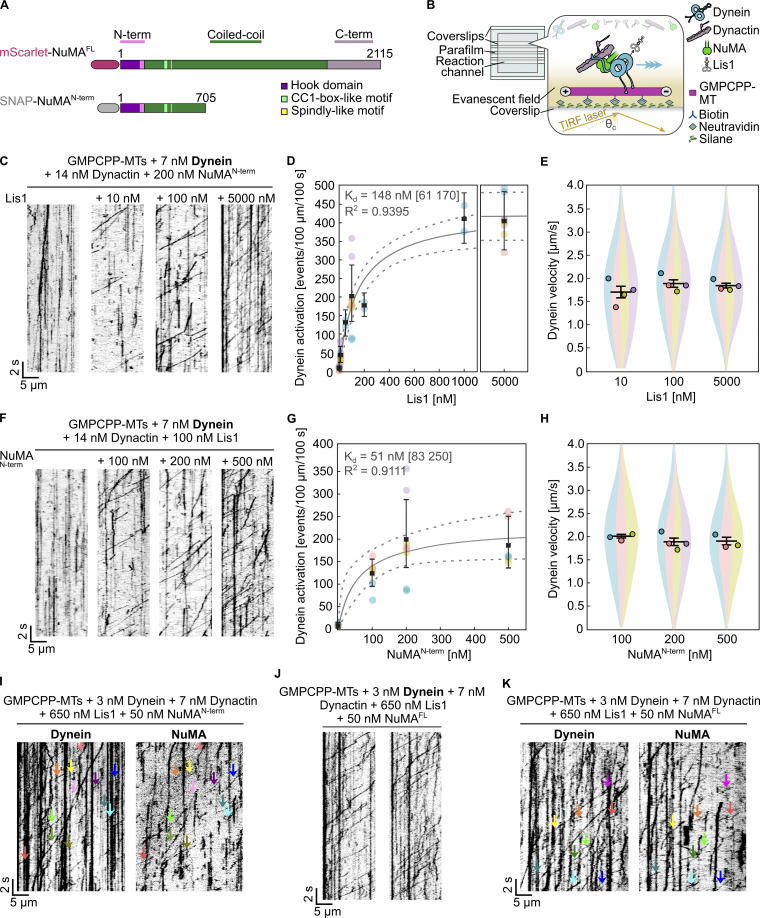
**NuMA is a dynein adaptor that requires dynactin and Lis1 to activate dynein motility. (A)** Schematic of mScarlet-tagged NuMA^FL^ and SNAP-tagged NuMA^N-term^ constructs, indicating the main structural parts of NuMA (N-terminal region, predicted coiled-coil, C-terminal tail) and the domains or motifs implicated in dynein/dynactin-binding (Okumura et al., 2018; Renna et al., 2020). **(B)** Schematic of microscopy flow chambers (left), with functionalized glass surface and components of dynein motility assays (right). **(C)** Representative TIRF microscopy kymographs showing the motility of mEGFP-dynein in the presence of dynactin and AF546-NuMA^N-term^ at different mCherry-Lis1 concentrations. Concentrations as indicated. **(D)** Processive run frequency of mEGFP-dynein (median ± 95% CI, estimated by bootstrapping). Each distinct color refers to a replicate; each data point represents the number of events in one field of view of one replicate; *n* = 99, 101, 27, 100, 29, 26, 103 microtubules. The grey curve represents a hyperbolic fit; apparent K_d_ and goodness-of-fit (expressed by R^2^) are indicated; dashed lines: 95% CI of the fit (estimated by bootstrapping). **(E)** Velocity distribution of processive mEGFP-dynein runs (mean of medians ± SEM). Each color refers to a replicate; each circle represents the median velocity of one replicate; *n* = 760, 3,751, 7,128, velocities; adjusted P values by Welch’s ANOVA with Holm-Sidak’s post-hoc test for multiple comparisons: 0.6121 (10 versus 100 nM), 0.6121 (10 versus 5,000 nM), and 0.6593 (100 versus 5,000 nM). Protein combination and concentrations in D and E as in C, and as indicated. **(F)** Representative kymographs showing the motility of mEGFP-dynein in the presence of dynactin and mCherry-Lis1 at different AF546-NuMA^N-term^ concentrations. Concentrations as indicated. **(G)** Processive run frequency of mEGFP-dynein (median ± 95% CI). Symbols and curve as in D; *n* = 103, 70, 100, 70 microtubules. **(H)** Velocity distribution of processive mEGFP-dynein runs (mean of medians ± SEM). Circles as in E; *n* = 1,585, 3,482, 2,649 velocities; adjusted P values by Welch’s ANOVA with Holm-Sidak’s post-hoc test for multiple comparisons: 0.7632 (100 versus 200 nM), 0.7084 (10 versus 500 nM), and 0.9235 (200 versus 500 nM). Protein combinations and concentrations in G, H as in F, and as indicated. **(I–K)** Representative kymographs showing the motility of (I) mEGFP-dynein (left) and AF647-NuMA^N-term^ (right) in the presence of dynactin and Lis1, (J) mEGFP-dynein in the presence of dynactin, Lis1, and mScarlet-NuMA^FL^, (K) mEGFP-dynein (left) and mScarlet-NuMA^FL^ (right) in the presence of dynactin and Lis1. Arrowheads of the same color indicate co-localization in the same processive events. All data refer to motility on surface-immobilized Atto647N-labeled GMPCPP-microtubules (MTs) in dynein microscopy buffer. All data are from at least three biological replicates. Experiments shown in C‒H and J were carried out at 30°C, and those shown in I and K at 18°C.

**Figure S1.**
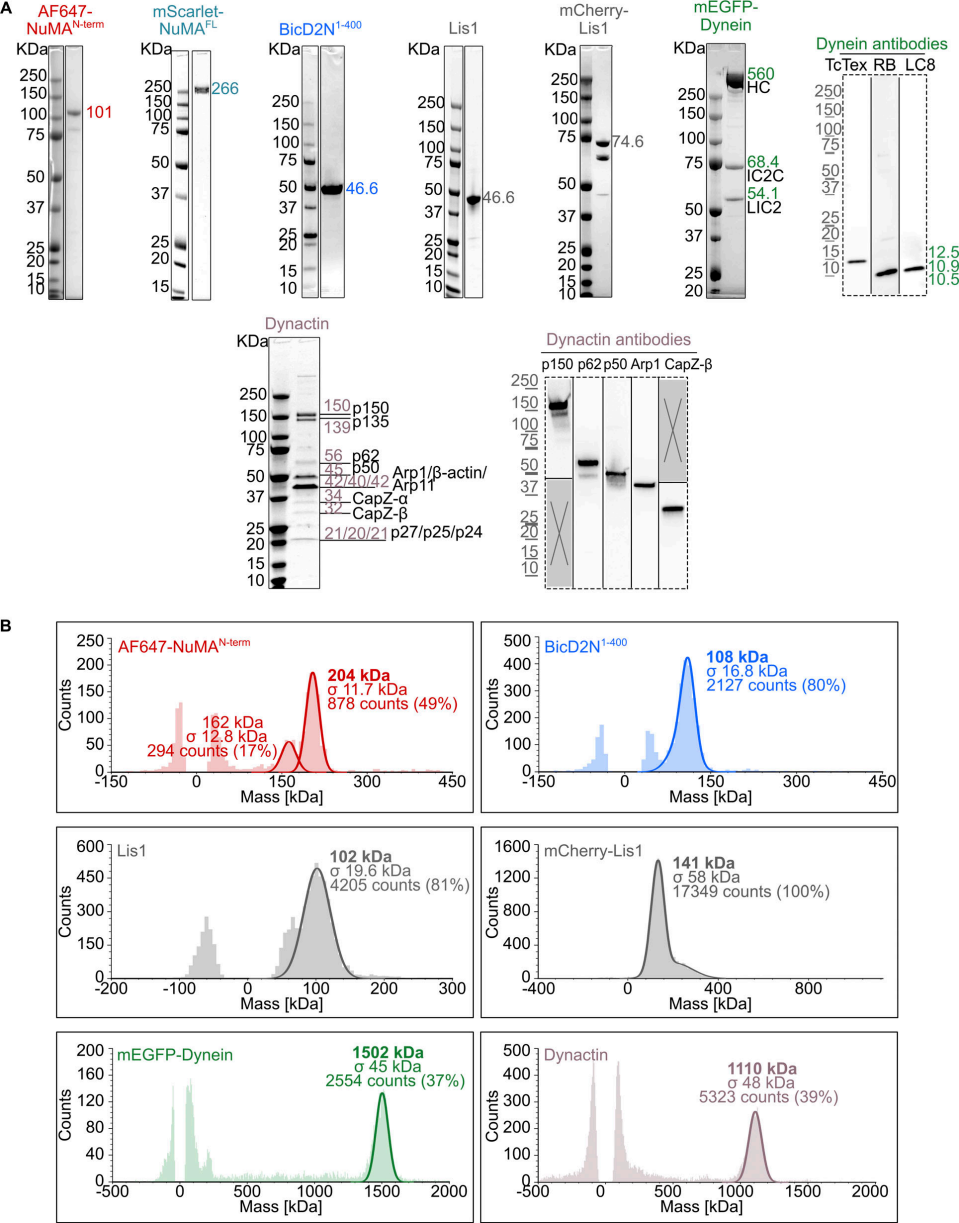
**Purified proteins used in dynein motility assays. (A)** Coomassie Blue-stained SDS-PAGE of the purified proteins. The expected molecular weight (kDa) of each purified protein is indicated. For dynein and dynactin complexes, the size and name of each subunit are specified. Western blots (dashed frames) were performed to detect the smallest dynein subunits (not visible on SDS-PAGE) and verify the identity of some dynactin SDS-PAGE bands. **(B)** Analysis of the oligomeric state of each purified protein by mass photometry. The molecular weight, with the associated standard deviation (σ), of the most abundant species present in each sample is indicated in the mass histograms. The symmetric parts of the histograms around mass 0 kDa represent the background signal of the buffer. For AF647-NuMA^N-term^, the 162 kDa peak may represent the oligomerized state of a minor contaminant of 80–85 kDa. Source data are available for this figure: SourceData FS1.

We purified a recombinant N-terminal fragment of human NuMA consisting of its first 705 amino acids (aa), fused to a SNAP-tag that was either labeled with Alexa Fluor 546 or 647 (AF546 or AF647-NuMA^N-term^, Fig. 1 A and Fig. S1 A). This NuMA construct was previously shown to immunoprecipitate dynein and dynactin (Kotak et al., 2012) and to recruit dynein to the cell cortex (Okumura et al., 2018). It contains a Hook domain that binds the dynein light intermediate chain in vitro (Renna et al., 2020). Due to the presence of part of NuMA’s predicted coiled-coil, NuMA^N-term^ was dimeric as demonstrated by mass photometry (Fig. S1 B). We also purified a recombinant human dynein complex with monomeric EGFP (mEGFP) fused to its heavy chain and porcine brain dynactin (Fig. S1 A), as described previously (Jha et al., 2017).

**Figure S2.**
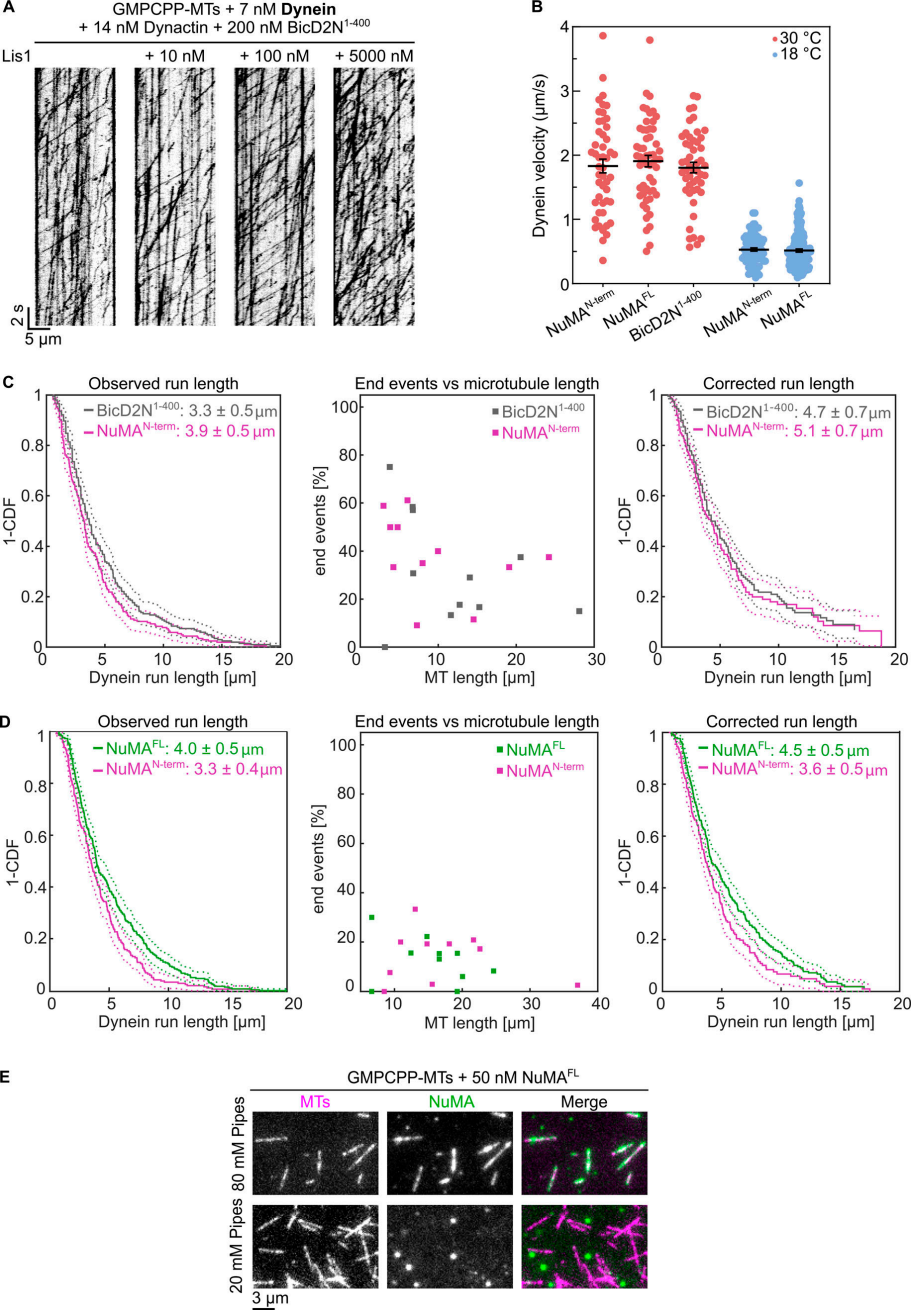
**Effect of Lis1, temperature, and adaptor identity on dynein motility. (A)** Representative kymographs showing the motility of mEGFP-dynein on surface-immobilized Atto647N-labeled GMPCPP-microtubules in the presence of dynactin and BicD2N^1–400^ at different mCherry-Lis1 concentrations. Concentrations as indicated. Lis1 stimulates BicD2N^1–400^-induced processive motility in a dose-dependent manner. **(B)** Velocity distributions of mEGFP-dynein processive runs (mean ± SEM) in the presence of either mScarlet-NuMA^N-term^, mScarlet-NuMA^FL^ or BicD2N^1–400^, as indicated. Each circle corresponds to the velocity of a single processive segment of a run; *n* = 49, 55, 50, 72, 166 velocities; adjusted P values by Welch’s ANOVA test with Holm-Sidak’s post-hoc test for multiple comparisons: 0.8246 (N-term versus FL, 30°C), 0.8429 (N-term versus BicD2N^1–400^, 30°C), 0.7750 (FL versus BicD2N^1–400^, 30°C); P value by Welch’s *t* test: 0.7228 (N-term versus FL, 18°C). Experiments performed with NuMA contained 3 nM mEGFP-dynein, 7 nM dynactin, 50 nM NuMA, 650 nM Lis1; experiments with BicD2N^1–400^ contained 7 nM mEGFP-dynein, 14 nM dynactin, 200 nM BicD2N^1–400^, 100 nM Lis1. Velocities measured at 18°C were approximately threefold lower than those measured at 30°C, consistent with the temperature effect previously reported (Hong et al., 2016; Ruhnow et al., 2017). **(C and D)** Comparison of run lengths for dynein activated by NuMA^N-term^ versus BicD2N^1–400^ (C) and by NuMA^N-term^ versus NuMA^FL^ (D). Left: survival probability (1-CDF) of all measured run lengths for NuMA^N-term^ (*n* = 204) and BicD2N^1‒400^-activated (*n* = 206) complexes. A large fraction of these processive events reach the microtubule end (NuMA^N-term^*n* end = 57; BicD2N^1–400^*n* end = 72); center: ratio of end-reaching events to total number of processive events per microtubule versus microtubule length; right: corrected survival probability of run length using the Kaplan-Meier estimator with end events being treated as right censored data points (Ruhnow et al., 2017). Survival probabilities are fitted with exponential function to estimate the run length; errors are approximated by $∆R=2R/\sqrt{N}$; dotted lines indicate the 95% CI of the survival probability. Experiments in C were performed at 7 nM mEGFP dynein, 14 nM dynactin, 100 nM mCherry-Lis1, and 200 nM AF546-NuMA^N-term^ or BicD2N^1‒400^. Experiments in D were performed at 3 nM mEGFP dynein, 7 nM dynactin, 650 nM Lis1, and 50 nM AF546-NuMA^N-term^ or mScarlet-NuMA^FL^. All experiments were carried out in dynein microscopy buffer. **(E)** Representative TIRF microscopy images showing the binding of 50 nM mScarlet-NuMA^FL^ to surface-immobilized Atto647N-labeled GMPCPP-microtubules in BRB80 (containing 80 mM Pipes, as in NuMA microscopy buffer) and BRB20 (containing 20 mM Pipes, as in dynein microscopy buffer), supplemented by 60 mM KCl. At 80 mM Pipes, NuMA binds all along the GMPCPP microtubules, as shown in Fig. 2 C. At 20 mM Pipes, NuMA’s solubility is reduced, as shown by numerous NuMA aggregates in solution, which impacts its ability to bind microtubules.

To test whether NuMA can act as a dynein adaptor, we immobilized GMPCPP-stabilized Atto647N-labeled microtubules on a glass surface, added AF546-NuMA^N-term^, mEGFP-dynein, and dynactin, and observed mEGFP-dynein by TIRF microscopy (Fig. 1 B). Under these conditions, we hardly ever observed processive motility events along microtubules (Fig. 1 C, left kymograph), in contrast to the typical behavior of dynein in the presence of dynactin and an adaptor (McKenney et al., 2014; Schlager et al., 2014). We found that the addition of purified human Lis1, which is known to relieve dynein’s autoinhibition (Qiu et al., 2019; Elshenawy et al., 2020; Htet et al., 2020; Marzo et al., 2020; Karasmanis et al., 2023), was required to trigger dynein to move processively in the presence of NuMA^N-term^ and dynactin. Lis1 increased the number of processive motility events in a dose-dependent manner (Fig. 1, C and D), similar to what can be observed with other adaptors, such as bicaudal D-related protein 1 (BicDR1) (Zhao et al., 2023) or protein bicaudal D homolog 2 N-terminus (BicD2N^1–400^) (Fig. S2 A), which however do not strictly require Lis1 (McKenney et al., 2014; Schlager et al., 2014; Schroeder and Vale, 2016; Redwine et al., 2017; Urnavicius et al., 2018; Canty et al., 2023). Increasing Lis1 concentration did not affect dynein velocity (Fig. 1 E), as previously also shown for BicD2 (Jha et al., 2017).

In the absence of NuMA^N-term^, as expected, Lis1 did not stimulate processive dynein motility because it is not an activating adaptor (Fig. 1 F, left kymograph). Increasing the concentration of NuMA^N-term^, while keeping the Lis1 concentration constant, increased the number of processive dynein motility events (Fig. 1, F and G) without affecting dynein velocity (Fig. 1 H). The average dynein velocity, measured at 30°C across all displayed conditions (Fig. 1, E and H), was ≈1.9 µm s^−1^. This is higher than reported in vitro velocities of mammalian dynein (Elshenawy et al., 2020; Htet et al., 2020; Canty et al., 2023; Zhao et al., 2023) due to the higher temperature in our experiments (Hong et al., 2016; Ruhnow et al., 2017) (Fig. S2 B). We observed no difference between dynein velocities or run lengths in the presence of NuMA^N-term^ or BicD2N^1–400^ under the same conditions (Fig. S2, B and C). Using a relatively low NuMA^N-term^ concentration to reduce fluorescence background and a relatively high Lis1 concentration allowed the visualization of AF647-NuMA^N-term^ transport by mEGFP-dynein, in agreement with NuMA’s activating dynein adaptor function (Fig. 1 I, arrowheads).

Next, we purified recombinant full-length human NuMA fused to the fluorescent protein mScarlet (Scarlet-NuMA^FL^) (Fig. S1 A) and tested its ability to stimulate processive dynein motility. We found that also NuMA^FL^ was able to activate dynein in the presence of both dynactin and Lis1 (Fig. 1 J), similar to what was observed with NuMA^N-term^. Dynein velocities and run lengths in the presence of NuMA^FL^ or NuMA^N-term^ were similar (Fig. S2, B and D). mScarlet-NuMA^FL^ could also be observed to be transported by mEGFP-dynein, again in agreement with NuMA’s activating adaptor function (Fig. 1 K, arrowheads). We did not attempt a quantitative comparison of the number of observed processive events promoted by NuMA^FL^ compared with NuMA^N-term^ given the considerably poorer solubility of NuMA^FL^ at the relatively low ionic strength conditions of these motility experiments (Fig. S2 E).

These results establish NuMA as a new dynein adaptor whose dynein processivity-stimulating activity depends more strongly on the additional presence of Lis1 than that of most other dynein adaptors.

### NuMA’s main microtubule-binding region is located close to its C-terminus

**Figure 2.**
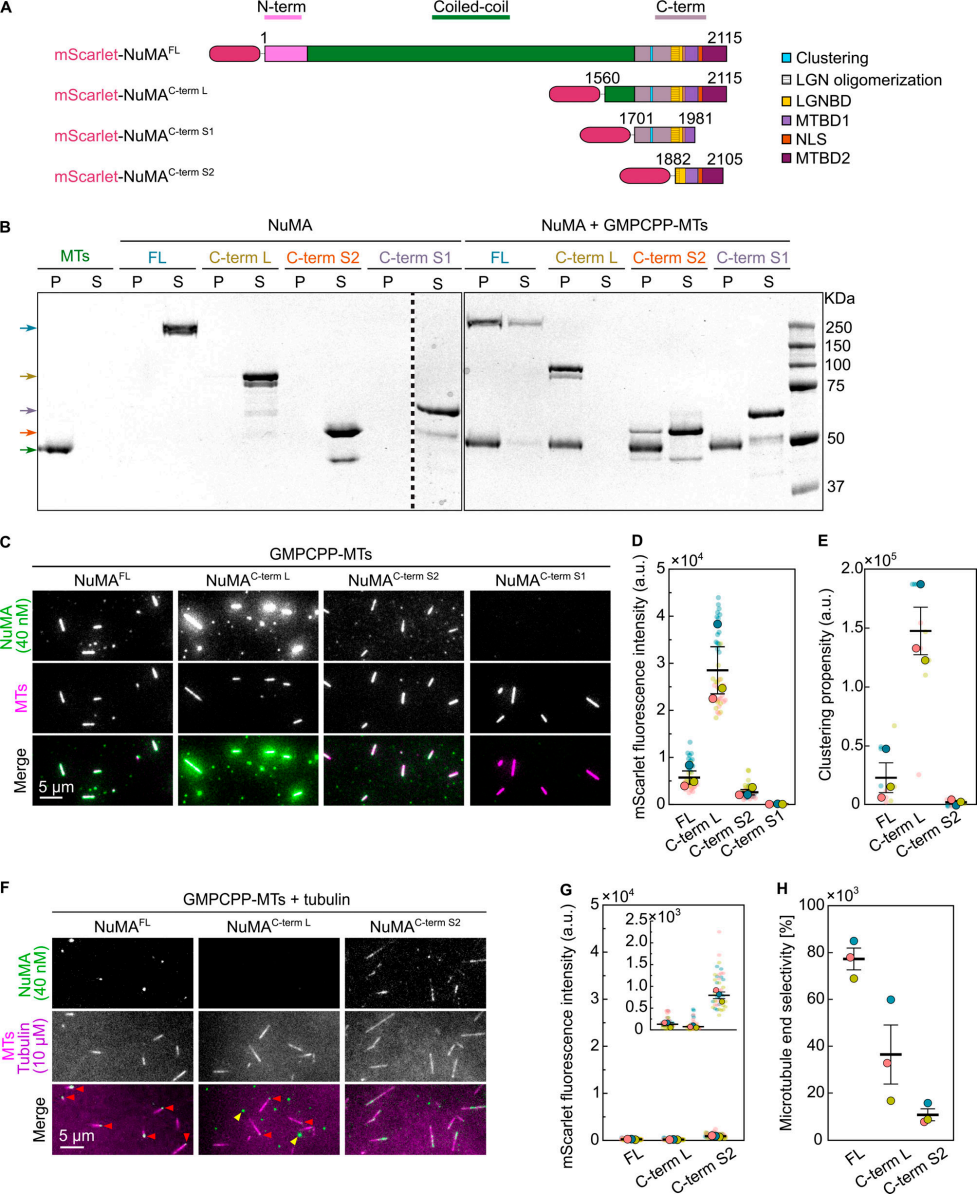
**NuMA’s microtubule-binding region is located close to its C-terminus. (A)** Schematic of mScarlet-tagged NuMA^FL^ and C-terminal fragments, indicating the main structural parts of NuMA and functional domains identified in the C-terminal region (microtubule-binding domain [MTBD] 1 [Du et al., 2002] and 2 [Gallini et al., 2016; Chang et al., 2017], clustering domain [Okumura et al., 2018], LGN oligomerization domain [Pirovano et al., 2019], LGN binding domain [LGNBD] [Pirovano et al., 2019], nuclear localization signal [NLS] [Chang et al., 2017]). **(B)** Coomassie Blue-stained SDS-PAGE showing the pellet (P) and supernatant (S) fractions of microtubule co-sedimentation assays. 1 µM mScarlet-tagged NuMA^FL^ or C-terminal fragments were incubated alone or with Atto647N-labeled GMPCPP-microtubules (0.5 µM polymerized tubulin) at 30°C for 15 min. Upon centrifugation, in the absence of microtubules, all proteins remained in the supernatant (left gel). When incubated with microtubules, they co-sedimented with the microtubule pellet (green arrow) at different degrees. NuMA^C-term L^ was found exclusively in the pellet; NuMA^FL^ predominantly in the pellet; NuMA^C-term S2^ partially in the pellet; NuMA^C-term S1^ entirely in the supernatant. Arrows indicate the expected molecular weight for each NuMA construct. **(C)** Representative TIRF microscopy images of 40 nM mScarlet-tagged NuMA constructs binding to surface-immobilized Atto647N-labeled GMPCPP-microtubules. **(D)** Background-corrected and microtubule length-corrected mScarlet intensity of the different NuMA constructs binding to microtubules as shown in C (mean of medians ± SEM). Each big circle represents the median value of one replicate; each small circle represents the intensity on a single microtubule; *n* = 45 microtubules for all conditions; P values by Welch’s *t* test: 0.0364 (FL versus C-term L), 0.1346 (FL versus C-term S1), 0.0530 (FL versus C-term S2). **(E)** Clustering propensity: sums of the background-corrected intensities of all outliers of the mScarlet NuMA intensity distributions for the different NuMA constructs shown in C nonspecifically bound to the glass surface (mean of medians ± SEM). Each big circle represents the median value of one replicate; each small circle represents the clustering at one surface area of one replicate; *n* = 9 areas for all conditions; adjusted P values by Welch’s ANOVA test with Holm-Sidak’s post-hoc test for multiple comparisons: 0.0297 (FL versus C-term L), 0.2367 (FL versus C-term S2), 0.0358 (C-term L versus C-term S2). **(F)** Representative TIRF microscopy images of 40 nM mScarlet-tagged NuMA constructs binding to surface-immobilized Atto647N-labeled GMPCPP-microtubules in the presence of 10 µM Atto647N-tubulin. Red arrowheads indicate selective end binding; yellow arrowheads indicate examples of NuMA^C-term L^ clusters nonspecifically adsorbed to the surface. **(G)** Background-corrected and microtubule length-corrected mScarlet intensity of the different NuMA constructs binding to microtubules in the presence of 10 µM Atto647N-tubulin as shown in E (mean of medians ± SEM). Symbols as in D; *n* = 45 microtubules for all conditions; adjusted P values by Welch’s ANOVA test with Holm-Sidak’s post-hoc test for multiple comparisons: 0.2825 (FL versus C-term L), 0.0143 (FL versus C-term S2), 0.0184 (C-term L versus C-term S2) **(H)** Microtubule end selectivity: percentage of microtubules showing mScarlet signal exclusively at one end, of all microtubules with an mScarlet signal, for mScarlet-NuMA constructs binding to microtubules in the presence of tubulin as shown in C (mean ± SEM). Each color represents a replicate; *n* = 126, 71, 161 microtubules; adjusted P values by Welch’s ANOVA test with Holm-Sidak’s post-hoc test for multiple comparisons: 0.1339 (FL versus C-term L), 0.0028 (FL versus C-term S2), 0.1732 (C-term L versus C-term S2). All data are from three biological replicates. All assays were executed in NuMA microscopy buffer. Source data are available for this figure: SourceData F2.

**Figure S3.**
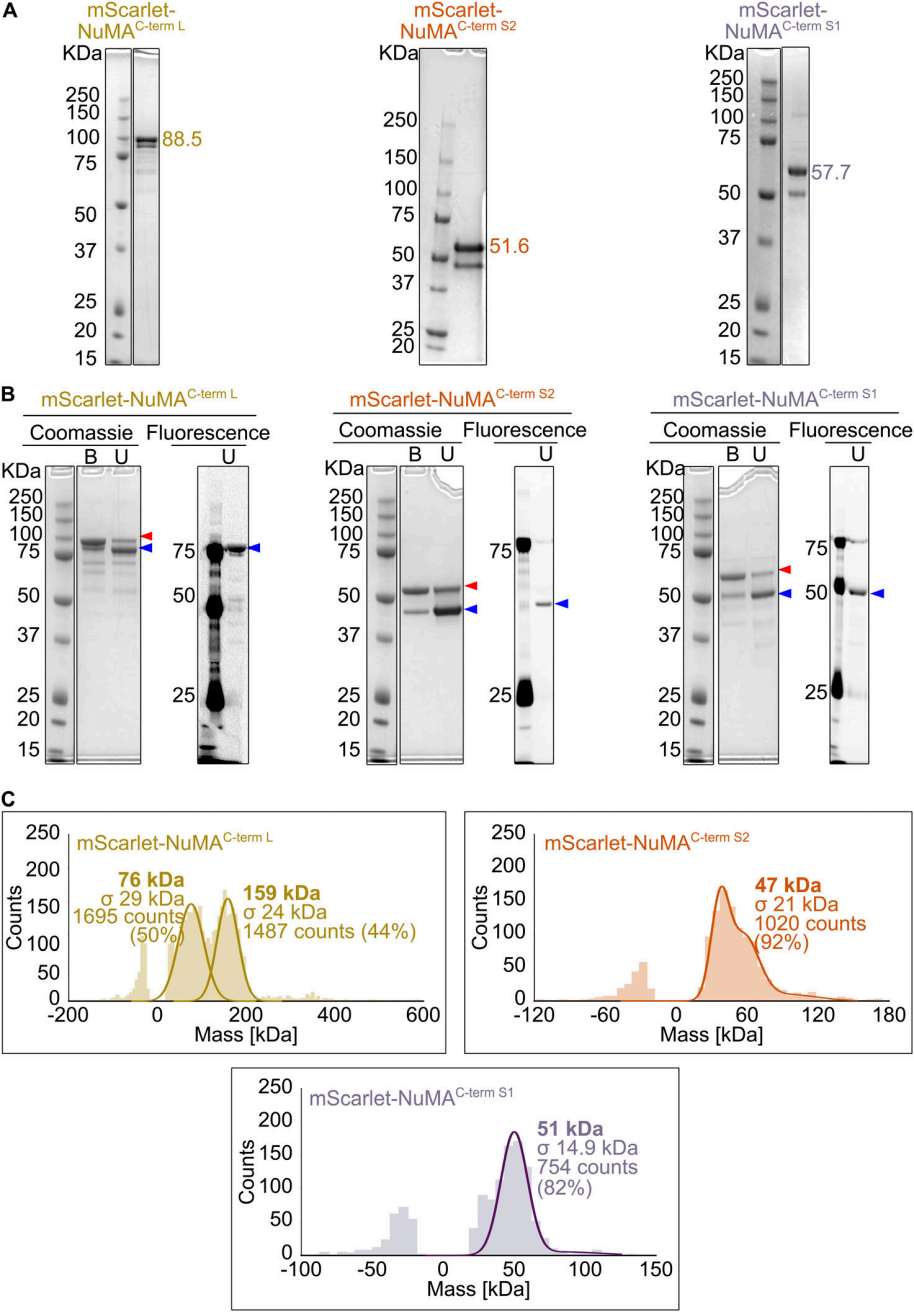
**Purified mScarlet-labeled NuMA C-terminal truncations. (A)** Coomassie Blue-stained SDS-PAGE of the purified NuMA C-terminal truncations. The expected molecular weight (kDa) of each NuMA construct is indicated; double bands are due to the mScarlet tag. **(B)** Comparison of Coomassie Blue staining and mScarlet fluorescent signal for samples described in A, unboiled (U), or boiled (B) in SDS sample buffer. When the protein is not boiled, it is mostly present in a state of lower apparent molecular weight (blue arrowhead), which corresponds to an mScarlet conformation preserving its fluorescence. On the contrary, upon boiling, the most abundant band is the one with higher apparent molecular weight (red arrowhead), which corresponds to a denatured mScarlet that does not fluoresce. A minor portion of protein is resistant to denaturation, which explains the double band pattern of all mScarlet-tagged NuMA proteins. **(C)** Analysis of the oligomeric state of mScarlet-tagged NuMA C-terminal constructs by mass photometry. Profiles indicate the calculated molecular weight, with the associated standard deviation (σ), of the most abundant species present in each sample. The symmetric parts of the histograms around 0 kDa represent the background signal of the buffer. In the case of NuMA^C-term L^, the two peaks of almost equal height represent a mixed population of monomers and dimers. Source data are available for this figure: SourceData FS3.

Next, we purified three recombinant C-terminal fragments of human NuMA fused to mScarlet (Fig. 2 A; and Fig. S3, A and B): (1) A long C-terminal fragment comprising aa 1560–2115 (NuMA^C-term L^), which contains part of the predicted coiled-coil and the entire C-terminal “tail” region previously reported to contain two MTBDs (Du et al., 2002; Gallini et al., 2016; Chang et al., 2017) and a clustering domain (Okumura et al., 2018). (2) A shorter fragment comprising aa 1882–2105 (NuMA^C-term S2^), which contains only part of the tail, including the reported MTBDs, but lacks the clustering domain (similar or identical to what was previously called NuMA-tail II [Nachury et al., 2001; Wiese et al., 2001; Haren and Merdes, 2002; Forth et al., 2014; Chang et al., 2017] or NuMA C-tail2 [Hueschen et al., 2017]). (3) Another short fragment comprising aa 1701–1981 (NuMA^C-term S1^), which contains also part of the tail, but lacks the most C-terminal reported microtubule-binding domain (previously named C-tail 1+2A [Hueschen et al., 2017]).

Using mass photometry, we observed that the longer NuMA^C-term L^ fragment was a mix of dimers and monomers, whereas both short fragments were monomers, as expected from their lack of any predicted coiled-coil (Fig. S3 C). We could not analyze the oligomerization state of NuMA^FL^, given its low concentration and the presence of detergent in its buffer.

Using a microtubule co-sedimentation assay, we observed that NuMA^FL^, NuMA^C-term L^, and NuMA^C-term S2^ were bound to GMPCPP-microtubules (Fig. 2 B). The binding of NuMA^C-term L^ was strongest, followed by NuMA^FL^ and NuMA^C-term S2^. NuMA^C-term S2^ binds more weakly to microtubules than the longer constructs, probably because it is monomeric. NuMA^C-term S1^ did not bind to microtubules under these conditions, in agreement with a previous in vitro study suggesting that the major microtubule-binding region in NuMA is at its very C-terminus (Chang et al., 2017). TIRF microscopy of the different mScarlet-NuMA constructs binding to surface-immobilized GMPCPP-microtubules confirmed the microtubule co-sedimentation results (Fig. 2 C). Quantifying the mScarlet-NuMA fluorescence intensity along microtubules confirmed that NuMA^C-term L^ bound best (Fig. 2 D), most likely due to oversaturating the microtubule lattice as a consequence of its pronounced clustering ability, as determined from quantifying the intensities of surface adsorbed NuMA (Fig. 2 E). Why the NuMA^C-term L^ fragment clusters more than NuMA^FL^ is currently unknown.

Next, we were interested in observing how the different NuMA constructs bind to GMPCPP-microtubules in the presence of tubulin (Fig. 2 F). NuMA binding to GMPCPP-microtubules was much reduced in the presence of free tubulin (Fig. 2 G). Remarkably, quantification of the mScarlet fluorescence at microtubule ends compared with the lattice indicated that NuMA^FL^ and NuMA^C-term L^ appeared to bind preferentially to one of the two microtubule ends (Fig. 2 F, red arrowheads, Fig. 2 H). NuMA^C-term L^ and to a lesser extent NuMA^FL^ were also observed to non-specifically adsorb to the surface as what appeared to be clusters of varying size (Fig. 2 F, yellow arrowheads), probably due to the presence of a clustering domain within these constructs (Okumura et al., 2018).

### NuMA’s microtubule-binding region preferentially binds to microtubule minus ends and prevents their growth

**Figure 3.**
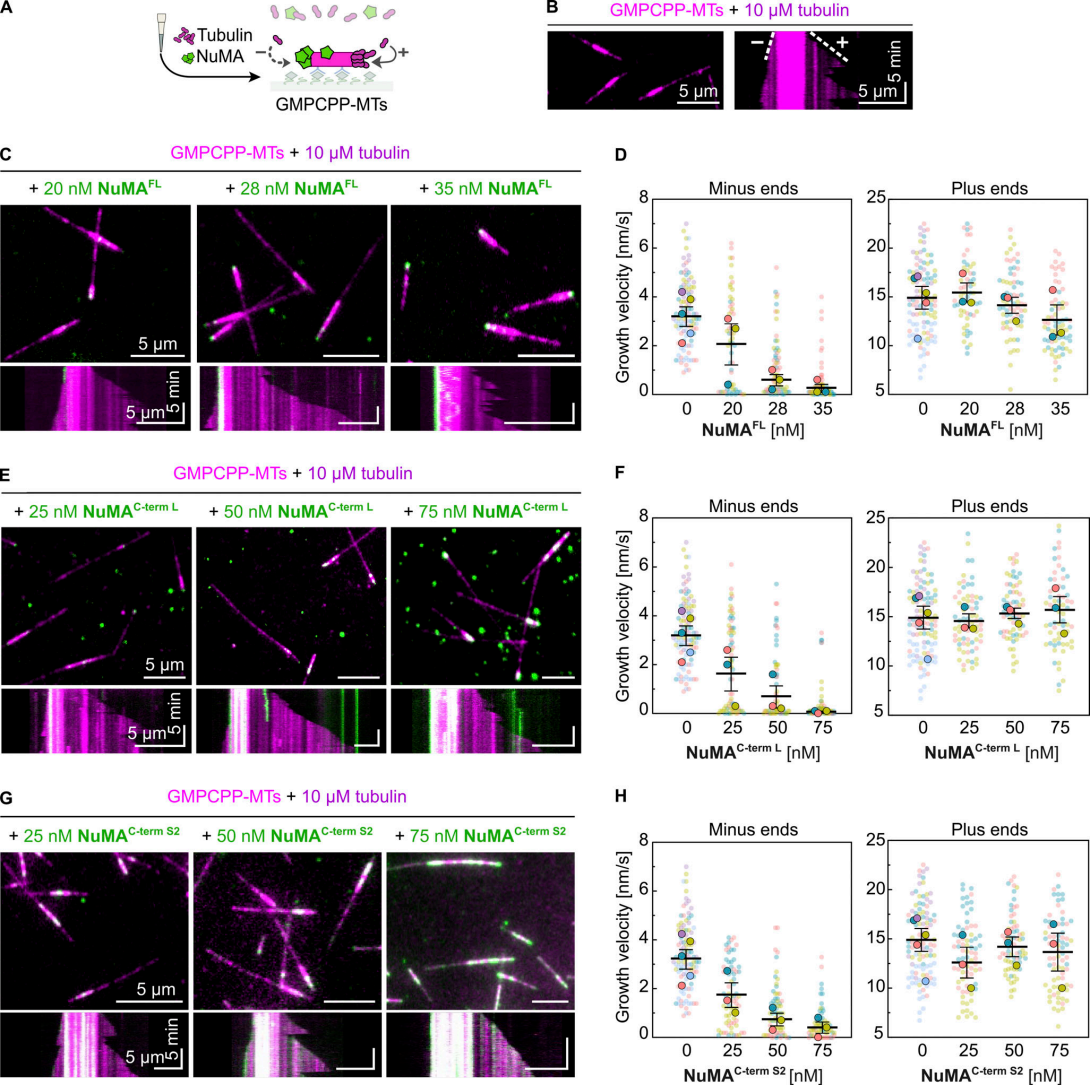
**NuMA’s microtubule-binding region preferentially binds to microtubule minus ends and prevents their growth. (A)** Schematic of a TIRF microscopy assay with fluorescent NuMA constructs and tubulin being flowed simultaneously into a channel containing surface-immobilized GMPCPP-microtubule seeds. **(B)** Representative TIRF microscopy image (left) and kymograph (right) showing minus and plus ends (dim magenta) dynamically elongating from a surface-immobilized Atto647N-labeled GMPCPP-seed (bright magenta) in the presence of Atto647N-tubulin. **(C, E, and G)** Representative TIRF microscopy images (top) and kymographs (bottom) showing the growth behavior of microtubule ends (dim magenta) elongating from surface-immobilized Atto647N-labeled GMPCPP-seeds (bright magenta) in the presence of Atto647N-tubulin and various concentrations of different mScarlet-tagged NuMA constructs (green). **(D, F, and H)** Growth velocity distributions of microtubule minus and plus ends related to the experiments shown in C, E, and G (mean of medians ± SEM). Each big circle represents the median velocity of one replicate; each small circle represents the growth velocity of one microtubule end segment; D: *n* = 109, 60, 69, 71 (left plot) and 109, 59, 68, 70 (right plot); adjusted P values: 0.3126, 0.0030, 0.0027 (left plot) and 0.8469, 0.8469, 0.6588 (right plot). F: *n* = 109, 72, 66, 67 (left plot) and 109, 72, 68, 65 (right plot); adjusted P values: 0.1334, 0.0188, 0.0041 (left plot) and 0.9642, 0.9642, 0.9642 (right plot). H: *n* = 109, 74, 70, 66 (left plot) and 109, 74, 70, 75 (right plot); adjusted P values: 0.0785, 0.0042, 0.0031 (left plot) and 0.6572, 0.8526, 0.8526 (right plot). All data are from at least three biological replicates; adjusted P values were calculated by Welch’s ANOVA test with Holm-Sidak’s post-hoc test for multiple comparisons; each condition was compared to the control at 0 nM NuMA. All experiments were performed in a NuMA microscopy buffer. All data are from three biological replicates.

To determine to which microtubule end NuMA binds with preference, we imaged NuMA^FL^ and the C-terminal NuMA fragments over time as microtubules elongated from the GMPCPP-microtubule “seeds” in the presence of free tubulin (Fig. 3 A). In the absence of NuMA, plus and minus ends can easily be distinguished by their different growth speeds (Fig. 3 B). Adding increasing concentrations of NuMA^FL^ slowed down the growth of the slower-growing minus ends in a dose-dependent manner (Fig. 3, C and D). In contrast, the growth of the faster-growing plus ends was largely unaffected in the studied concentration range. At the highest tested concentration, minus-end growth was completely prevented, with NuMA^FL^ accumulating selectively to these ends, demonstrating that NuMA has a microtubule minus-end binding preference. Only a minor decrease in velocity was observed for the growth of plus ends at the highest NuMA^FL^ concentration. At the higher concentrations, NuMA^FL^ was also observed to bind with some preference to the immobilized GMPCPP-seed.

**Figure S4.**
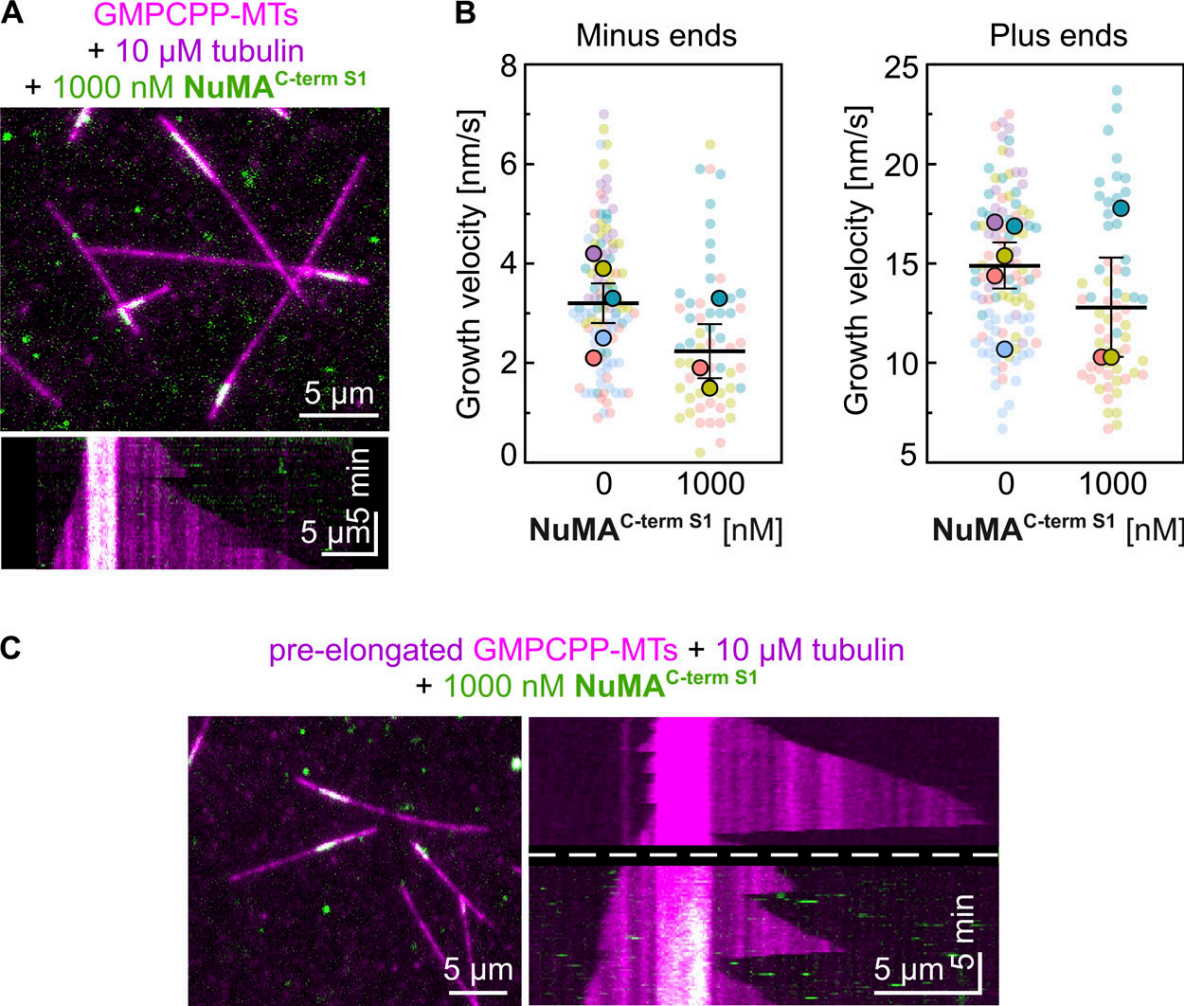
**Effect of NuMA** ^**C-term S1**^ **at a high concentration on microtubule dynamics. (A)** Representative TIRF microscopy image (top) and kymograph (bottom) showing the growth behavior of microtubule ends (dim magenta) elongating from surface-immobilized Atto647N-labeled GMPCPP-seeds (bright magenta) in the presence of Atto647N-tubulin and mScarlet-NuMA^C-term S1^ (green). **(B)** Growth velocity distributions of minus and plus ends of microtubules under conditions explained in A (mean of medians ± SEM). Each big circle represents the median velocity of one replicate; each small circle represents the growth velocity of one microtubule end segment; *n* = 101, 58 (left plot) and 109, 63 (right plot); P values by Welch’s *t* test: 0.2241 (left plot), 0.5035 (right plot). **(C)** Representative TIRF microscopy image (left) and kymograph (right) showing the growth behavior of microtubule ends (dim magenta) elongating from surface-immobilized Atto647N-labeled GMPCPP-seeds (bright magenta) in the presence of Atto647N-tubulin, before (above the dashed line) or after (below the dashed line) the addition of mScarlet-NuMA^C-term S1^ (green).

Similar behavior was observed for NuMA^C-term L^ (Fig. 3, E and F) and NuMA^C-term S2^ (Fig. 3, G and H). NuMA^C-term S2^ was additionally seen to bind weakly all along GDP-microtubule segments, in agreement with enhanced binding of a similar construct along mitotic spindle microtubules in cells (Hueschen et al., 2017). In contrast, NuMA^C-term S1^ did not exert a clear effect on microtubule dynamics, not even at elevated concentrations, when it began to weakly bind to GMPCPP-seeds (Fig. S4, A and B).

Taken together, these results show that NuMA’s main microtubule-binding domain, located at its C-terminus, is required for selective minus-end growth inhibition and that NuMA^C-term S2^ is sufficient to exert this effect, even though longer NuMA constructs appear to act more strongly.

### NuMA caps and stabilizes dynamic microtubule minus ends

**Figure 4.**
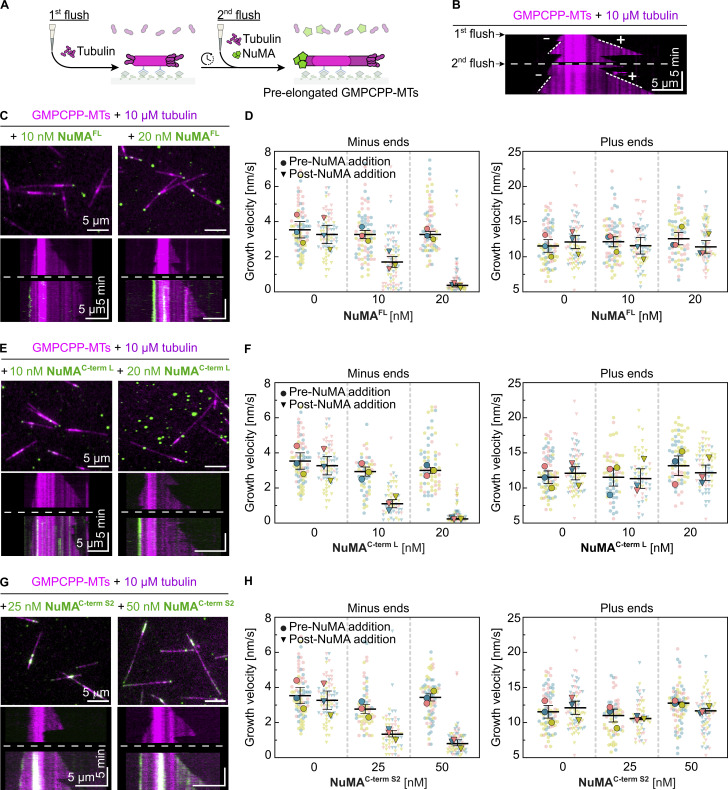
**NuMA caps and stabilizes dynamic microtubule minus ends. (A)** Schematic of a two-flush TIRF microscopy assay: fluorescent tubulin is flowed into a channel containing surface-immobilized GMPCPP-seeds (first flush); microtubules are allowed to elongate from GMPCPP-seeds for ≈10 min; and tubulin is re-added together with fluorescent NuMA constructs (second flush). **(B)** Representative kymograph showing a control experiment with microtubule minus and plus ends (dim magenta) dynamically elongating from a surface-immobilized Atto647N-labeled GMPCPP-seed (bright magenta) in the presence of 10 µM Atto647N-tubulin; the dashed line marks the second flush of tubulin. **(C, E, and G)** Representative TIRF microscopy images (top) and kymographs (bottom) showing the growth behavior of microtubule ends (dim magenta) elongating from surface-immobilized Atto647N-labeled GMPCPP-seeds (bright magenta) in the presence of Atto647N-tubulin, before (above the dashed line) or after (below the dashed line) the addition of different mScarlet-tagged NuMA constructs (green) at different concentrations. Related to Video 1. **(D, F, and H)** Growth velocity distributions of minus and plus ends elongating from GMPCPP-seeds in the presence of 10 µM Atto647N-tubulin before (circles) and after (triangles) the addition of different mScarlet-tagged NuMA constructs at different concentrations (mean of medians ± SEM). Each big symbol represents the median velocity of one replicate and each small symbol represents the growth velocity of one microtubule end segment; D: *n* = 72, 73, 67; P values: 0.0572, 0.0111, 0.0012 (left plot) and 0.1221, 0.4972, 0.0008 (right plot). F: *n* = 72, 51, 66 (left plot) and 72, 53, 66 (right plot); P values: 0.0572, 0.0196, 0.0027 (left plot) and 0.1221, 0.9029, 0.4875 (right plot). H: *n* = 72, 57, 72 (left and right plots); P values: 0.0572, 0.0038, 0.0175 (left plot) and 0.1221, 0.6881, 0.1384 (right plot). All data are from three biological replicates; P values were calculated by paired *t* test comparing the “pre-NuMA addition” and “post-NuMA addition” for each condition. All experiments were performed in NuMA microscopy buffer.

To exclude that minus-end recognition by NuMA is a GMPCPP-microtubule-specific effect, we performed microtubule pre-elongation experiments (Fig. 4, A and B). We first allowed dynamic microtubules to grow from surface-immobilized GMPCPP-seeds in the presence of tubulin for ≈10 min, before adding NuMA while keeping the tubulin concentration constant. Also here, NuMA^FL^ bound preferentially to microtubule minus ends, stopping their growth. Moreover, it stabilized these minus ends, preventing catastrophe after growth stopped, making them static. In contrast, plus-end growth was again mostly unaffected (Fig. 4, C and D; and Video 1). NuMA^C-term L^ and NuMA^C-term S2^ exhibited similar effects (Fig. 4, E–H). In agreement with our previous observations, NuMA^C-term S1^ did not bind to dynamic microtubule ends (Fig. S4 C).

**Video 1.** **NuMA caps and stabilizes dynamic microtubule minus ends.** Microtubules elongate from surface-immobilized Atto647N-labeled GMPCPP-seeds (bright magenta), in the presence of 10 µM Atto647N-labeled tubulin (dim magenta). Minus ends can be distinguished from plus ends as they display slower growth. After ≈10 min of imaging, 10 µM fluorescent tubulin is flowed again into the channel (white frames), together with 20 nM mScarlet-NuMA^FL^ (green), which localizes selectively at the pre-elongated microtubule minus ends, arresting their growth and preventing catastrophe. The timestamp refers to mm:ss. Acquisition rate: 10 fps, display rate: 27 fps. Related to Fig. 4.

In conclusion, NuMA preferentially binds to dynamic microtubule minus ends, stabilizing and capping them at nanomolar NuMA concentrations. These results establish NuMA as a new autonomous minus end capper.

### γTuRC prevents NuMA accumulation at microtubule minus ends

**Figure 5.**
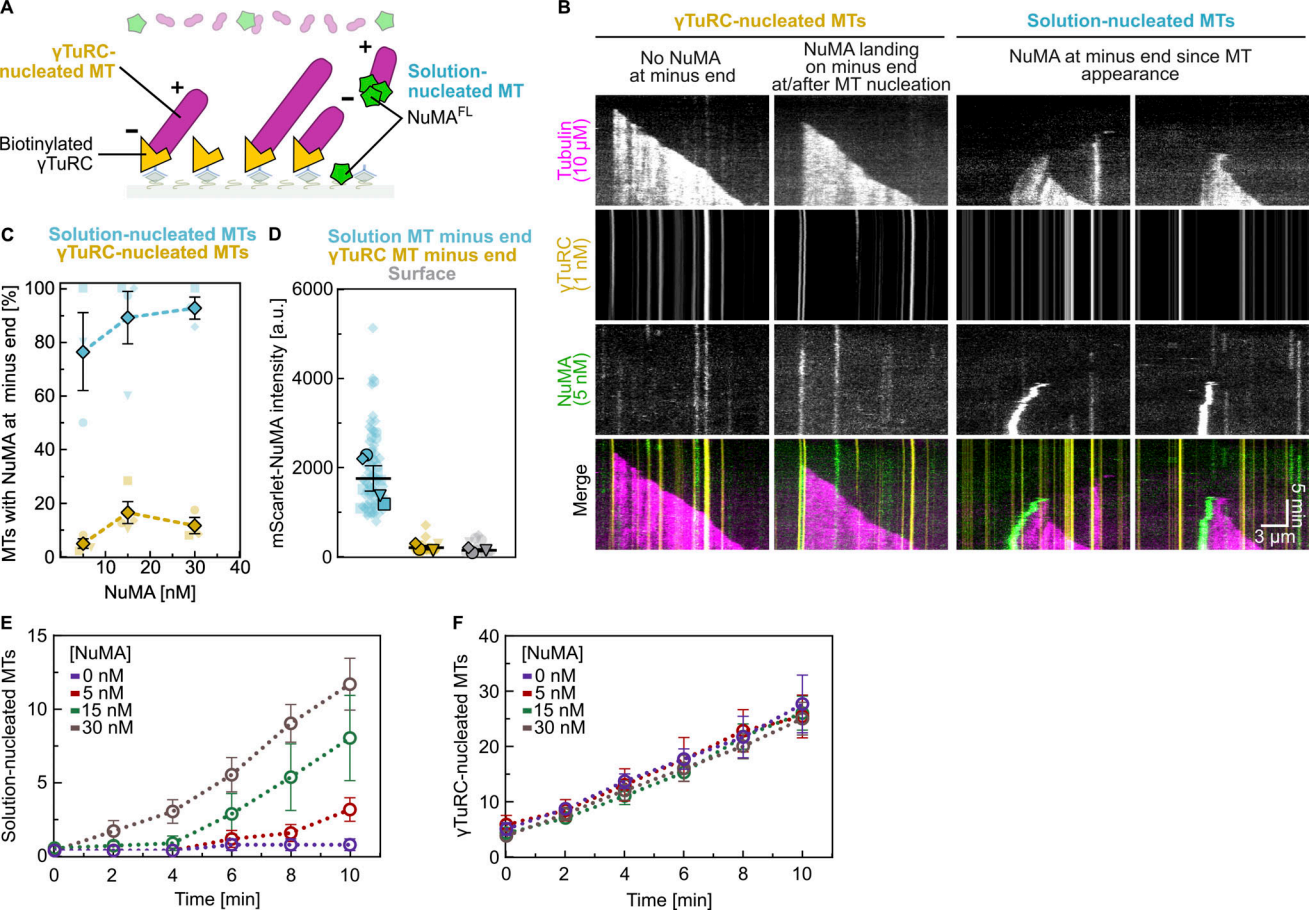
**γTuRC prevents NuMA accumulation at microtubule minus ends. (A)** Schematic of a γTuRC nucleation assay performed in the presence of mScarlet-NuMA^FL^. **(B)** Representative TIRF microscopy kymographs showing microtubules nucleated by surface-immobilized mBFP-γTuRC, and spontaneously nucleated microtubules in solution in the presence of 10 µM Atto647-tubulin and 5 nM mScarlet-NuMA^FL^. γTuRC and solution-nucleated microtubules can be distinguished because the latter diffuse on the surface. γTuRC-nucleated microtubules only occasionally display weak NuMA fluorescence at their minus ends (second kymograph), whereas the minus ends of solution-nucleated microtubules frequently show intense NuMA signals (third and fourth kymographs). **(C)** Frequency of microtubule minus end localization of NuMA on γTuRC-nucleated and spontaneously nucleated microtubules in the presence of 10 µM Atto647-tubulin and 5, 15, and 30 nM mScarlet-NuMA^FL^ (mean ± SEM); each diamond represents the mean frequency of each condition, each distinct small symbol represents the frequency of a single replicate within a condition; bottom *n* = 80, 101, 104 microtubules; top *n* = 21, 54, 52 microtubules. For γTuRC-nucleated microtubules, cases in which NuMA localization at the minus end coincided with NuMA/γTuRC co-localization prior to nucleation were excluded from the analysis. **(D)** Background-corrected maximum intensity of mScarlet-NuMA^FL^ at 30 nM localizing to either γTuRC-nucleated microtubule minus ends, spontaneously nucleated microtubule minus ends, or at randomly chosen microtubule-free surface areas (mean of medians ± SEM), in the presence of 10 µM Atto647-tubulin. Each big symbol represents the median of one replicate; each small symbol represents the intensity at one microtubule minus end or surface area; each replicate is represented by a distinct symbol shape (circle, diamond, triangle, square); *n* = 68, 18, 12 intensities; adjusted P values by Welch’s ANOVA test with Holm-Sidak’s post-hoc test for multiple comparisons: 0.0295 (solution versus γTuRC), 0.0295 (solution versus surface), 0.3793 (γTuRC versus surface). **(E and F)** Plots showing an increase of the solution-nucleated (E) and γTuRC-nucleated (F) microtubule number over time, in the presence of 0–30 nM mScarlet-NuMA^FL^ (mean ± SEM). NuMA promotes spontaneous nucleation of microtubules in solution in a dose-dependent manner (E), without exerting any effect on γTuRC-mediated nucleation (F). Experiments were performed in γTuRC microscopy buffer. All data are from at least three biological replicates.

Most microtubules in eukaryotic cells are nucleated by the γ-tubulin ring complex (γTuRC), which naturally caps their minus ends from the start of the growth (Zheng et al., 1995; Moritz et al., 2000; Consolati et al., 2020; Wieczorek et al., 2021; Rai et al., 2024). We, therefore, tested whether NuMA could bind to the minus ends of γTuRC-nucleated microtubules. We immobilized purified mBFP-labeled and biotinylated human γTuRC on a functionalized glass surface and observed by TIRF microscopy how microtubules were nucleated and grew in the presence of NuMA^FL^ (Fig. 5 A). Most microtubules were nucleated by γTuRC, but some were also nucleated spontaneously in solution.

γTuRC-nucleated microtubules grew only at their plus end, while their minus end was anchored to surface-immobilized γTuRC. In contrast, spontaneously nucleated microtubules in solution suddenly “landed” on the surface and moved diffusively. At the highest NuMA concentration tested, strong NuMA accumulation could be detected at the minus ends of ≈93% of these spontaneously nucleated microtubules (Fig. 5 B, solution-nucleated MTs, Fig. 5 C), whereas only ≈12% of the γTuRC-nucleated microtubules showed apparent NuMA localization at the minus end after the microtubule had nucleated (Fig. 5 B, γTuRC-nucleated MTs right, Fig. 5 C). This percentage is similar to that expected for random apparent co-localization due to nonspecific surface adsorption of NuMA (Materials and methods). This explains the low NuMA intensity that is much lower than at free minus ends and very similar to that of surface adsorbed NuMA (Fig. 5 D). γTuRC was never removed from minus ends by the presence of NuMA, in clear contrast to γTuRC removal by microtubule minus-end binding CAMSAP proteins (Rai et al., 2024). We, therefore, consider it unlikely that a minority of minus ends of γTuRC-nucleated microtubules weakly bind NuMA and conclude that γTuRC protects minus ends from NuMA accumulation.

We also observed that NuMA stimulated spontaneous nucleation of microtubules in solution in a dose-dependent manner, probably by stabilizing minus ends (Fig. 5 E), in clear contrast to NuMA having no effect on the efficiency of microtubule nucleation by γTuRC (Fig. 5 F), in agreement with γTuRC and NuMA competing for minus-end binding (Fig. 5, B and C). NuMA has not been reported to promote microtubule nucleation in mitotic cells, probably, as our results suggest, because microtubule nucleation is essentially entirely controlled by γTuRC-dependent pathways.

### NuMA gradually accumulates at the minus ends of enzymatically γTuRC-uncapped microtubule minus ends

**Figure 6.**
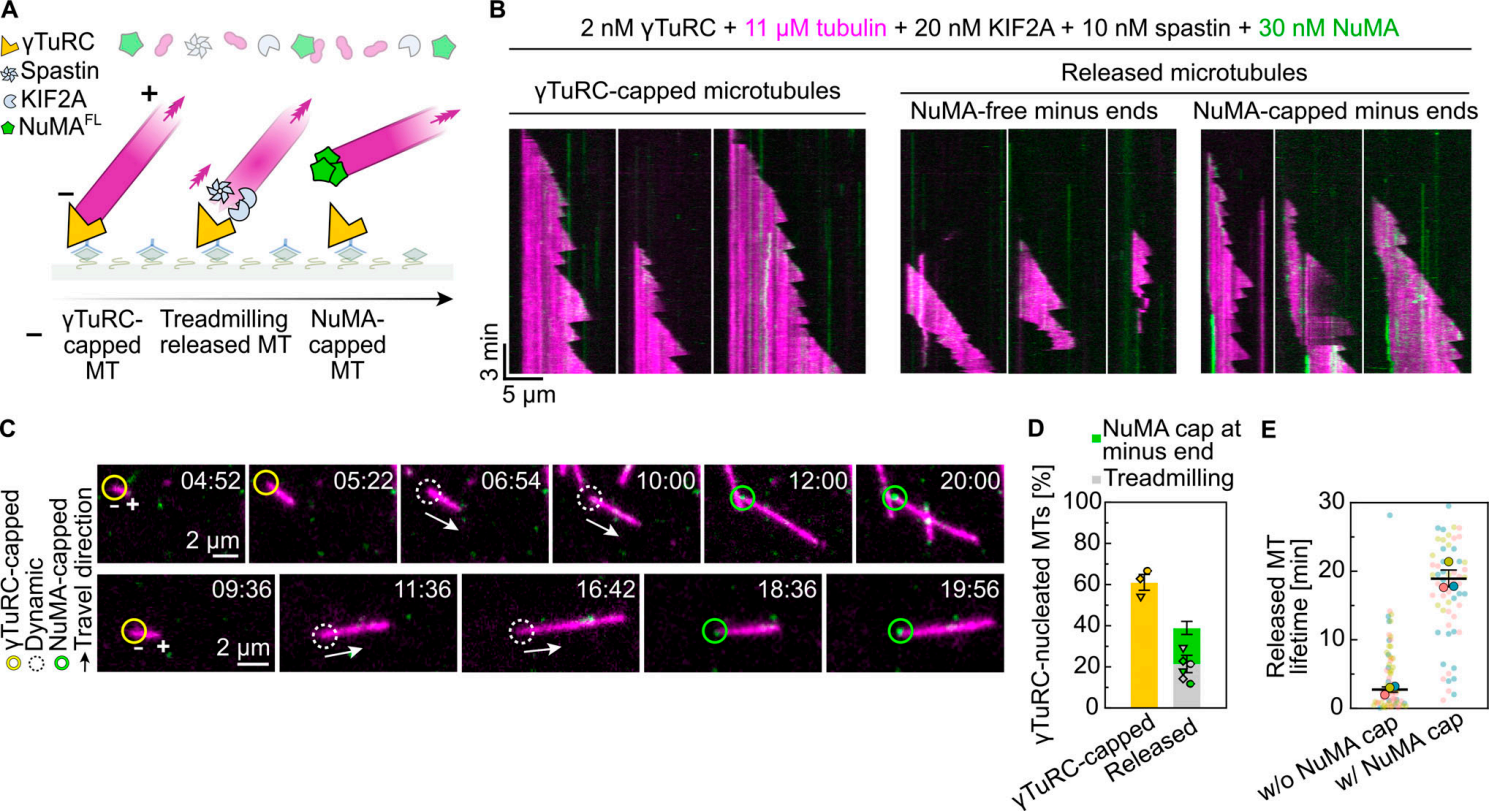
**NuMA gradually accumulates at the minus ends of enzymatically γTuRC-uncapped microtubule** **s** **. (A)** Schematic of a γTuRC nucleation assay performed in the presence of KIF2A, spastin, and mScarlet-NuMA^FL^. The combined action of spastin and KIF2A triggers γTuRC release, which is followed by microtubule treadmilling and minus end capping by NuMA. **(B)** Representative TIRF microscopy kymographs showing microtubules nucleated by surface-immobilized mBFP-γTuRC in the presence of 11 µM Atto647-tubulin, 20 nM KIF2A, 10 nM spastin, and 30 nM mScarlet-NuMA^FL^. γTuRC-capped microtubule minus ends are stabilized, while released microtubule minus ends typically depolymerize under the action of KIF2A, which also increases the catastrophe frequency at the plus end, often resulting in early microtubule disappearance. After some time, NuMA can cap the minus ends of released microtubules, arresting treadmilling and increasing their lifetimes. **(C)** Representative TIRF microscopy time course images of microtubule minus ends stabilized by γTuRC (yellow circles) undergoing γTuRC release, consequential treadmilling (white dashed circles), and eventually NuMA capping (green circles). Protein concentrations as in B; timestamps refer to mm:ss; related to Video 2. **(D)** Frequency of different γTuRC-nucleated microtubule populations (mean ± SEM). Each distinct symbol represents a replicate; protein concentrations as in B; *n* = 349 γTuRC-nucleated microtubules. **(E)** Lifetime of γTuRC-nucleated microtubules following their release, in the absence or presence of NuMA capping their minus ends (means of medians ± SEM). Each big circle represents the median value of one replicate, each small symbol represents the lifetime of one microtubule of one replicate; *n* = 133 γTuRC released microtubules; P value by Welch’s *t* test: 0.0030. Experiments were performed in γTuRC release buffer. All data are from three biological replicates.

We showed recently that the severing enzyme spastin and the microtubule depolymerase KIF2A can release γTuRC from the microtubule minus ends (Henkin et al., 2023). To test whether the removal of γTuRC allows NuMA accumulation at the minus ends of initially γTuRC-nucleated microtubules, we added full-length NuMA to γTuRC-nucleated microtubules in the presence of these two enzymes (Fig. 6 A). Around 40% of the microtubules were released from surface-immobilized γTuRC. Roughly half of these released microtubules treadmilled, as a consequence of KIF2A-mediated minus-end depolymerization and continued plus-end growth. The other half of the released microtubules displayed a strong accumulation of NuMA at their minus ends after some time, which stopped their KIF2A-driven minus-end depolymerization (Fig. 6, B–D and Video 2). NuMA accumulation at minus ends extended the microtubule lifetime due to minus-end stabilization (Fig. 6 E).

**Video 2.** **NuMA gradually accumulates at the minus ends of enzymatically γTuRC-uncapped microtubule** **s** **.** Microtubules nucleated by 2 nM of surface-immobilized mBFP-γTuRC in the presence of 11 µM Atto647-tubulin (magenta), 20 nM KIF2A, 10 nM spastin, and 30 nM mScarlet-NuMA^FL^ (green) displaying different behaviors. Minus ends that remain anchored to γTuRC (yellow arrow) are stabilized; minus ends that are released from γTuRC by enzymatic severing (white arrows) undergo KIF2A-mediated depolymerization, leading to microtubule treadmilling and often the complete disappearance of the microtubule (left white arrow); minus ends of treadmilling microtubules can be capped and stabilized by NuMA (green arrow), which lands on them only after some time. The timestamp refers to mm:ss. Acquisition rate: 2 fps, display rate: 75 fps. Related to Fig. 6.

These results indicate that NuMA competes for minus-end binding with KIF2A, which has previously been shown to accumulate at depolymerizing microtubule minus ends (Henkin et al., 2023). Our results show that directly after γTuRC-uncapping, typically KIF2A drives microtubule minus-end depolymerization, but later becomes replaced by NuMA, which then caps the minus end.

### NuMA binds to the minus ends of laser-ablated microtubules

**Figure 7.**
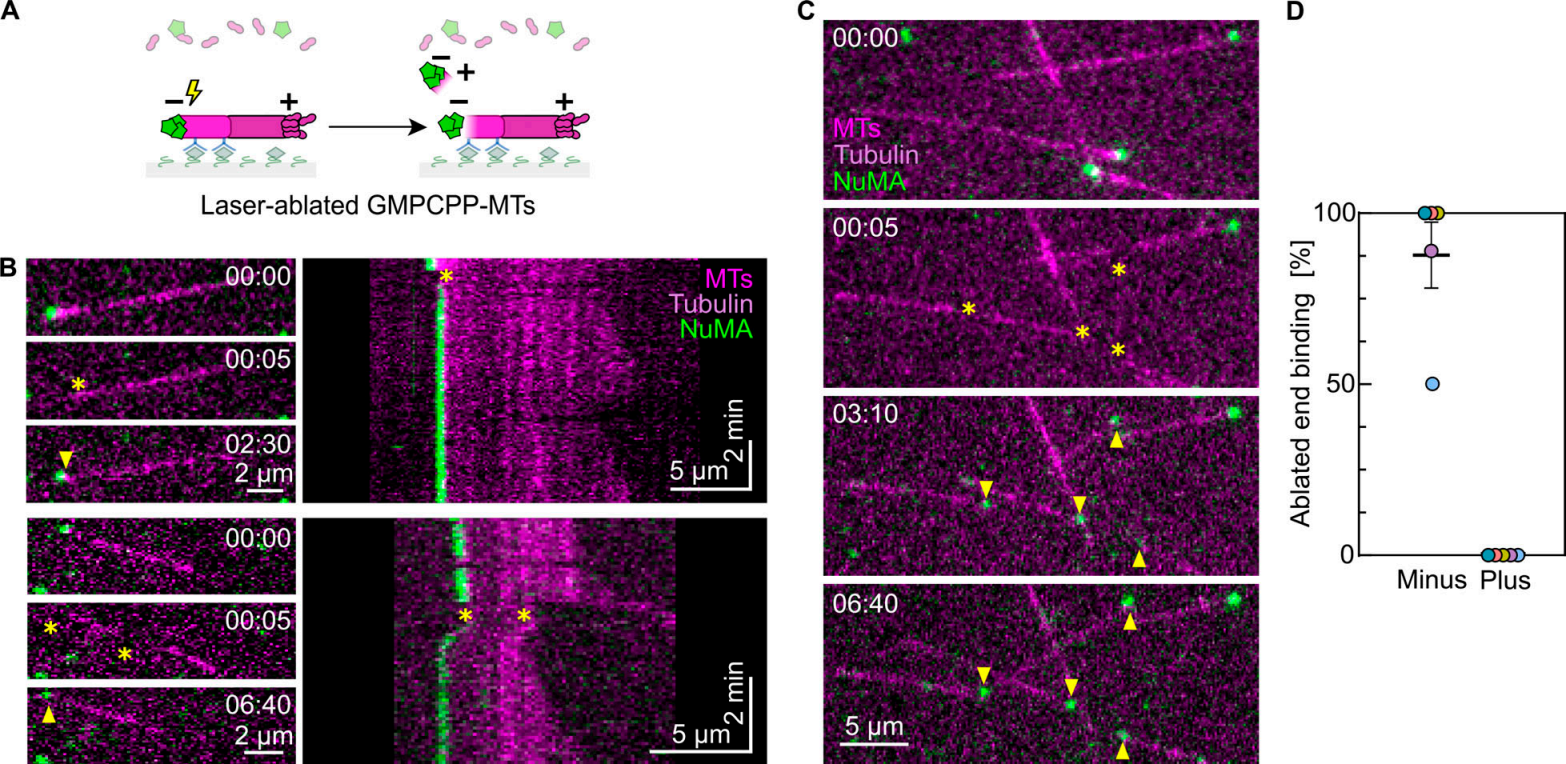
**NuMA binds to the minus ends of** **laser-ablated** **microtubules. (A)** Schematic of a TIRF microscopy assay with microtubules elongating from surface-immobilized GMPCPP-seeds being severed by laser ablation in the presence of mScarlet-NuMA^FL^. **(B and C)** Representative TIRF microscopy time course images of a microtubule elongating from a surface-immobilized Atto647N-labeled GMPCPP-seed (magenta) in the presence of 40 nM mScarlet-NuMA^FL^ (green) and 10 µM Atto647N-tubulin (magenta). Upon severing of the microtubule by laser ablation (asterisks), NuMA binds selectively to the newly generated minus end (arrowheads). Timestamps refer to mm:ss. **(B)** Ablation performed at one (top panel) or two (bottom panel) sites close to the microtubule minus end generates short segments that diffuse away in solution, thus only the minus end of the longest fragment which stays anchored to the surface can be observed. **(C)** Ablation performed farther from the microtubule minus end produces longer fragments that remain attached to the surface, allowing visualization of both new plus and minus ends. Related to Video 3. **(D)** Frequency of NuMA localization to freshly generated plus and minus ends after laser ablation in the presence of 40 nM mScarlet-NuMA^FL^ and 10 µM Atto647N-tubulin (mean ± SEM, five biological replicates). Each color represents a replicate; *n* = 54 ablation sites. All experiments were performed in NuMA microscopy buffer.

To test how NuMA binds to acutely generated new microtubule minus ends in the absence of any uncapping proteins, we tested whether and how NuMA binds to new microtubule minus ends generated by microtubule severing using laser ablation in the absence of spastin or KIF2A (Fig. 7 A). After laser cutting of dynamic microtubules growing from surface-immobilized GMPCPP-seeds, NuMA bound to and accumulated at the newly generated minus ends (Fig. 7, B and C). When the cut was executed close to the microtubule minus end, the severed short microtubule minus segment detached from the surface, diffusing away, and binding to the new minus end could be observed (Fig. 7 B). Cuts performed farther from the microtubule end produced longer microtubule segments that remained close to the surface, allowing us to observe both freshly generated plus and minus ends (Fig. 7 C and Video 3). NuMA was never observed to bind to new plus ends, whereas ≈83% of the ablation-generated minus ends had NuMA bound (Fig. 7 D).

**Video 3.** **NuMA binds to the minus ends of** **laser-ablated** **microtubules.** Microtubules elongated from surface-immobilized Atto647N-labeled GMPCPP-seeds (bright magenta), in the presence of 10 µM of Atto647N-labeled tubulin (dim magenta) and 40 nM mScarlet-NuMA^FL^ (green). Minus ends can be distinguished from plus as they are capped by NuMA and are not growing. Upon severing of the microtubules by laser ablation at multiple sites (asterisks), the longer microtubule fragments that remain attached to the surface allow the observation of both newly generated plus and minus ends. NuMA localizes and accumulates preferentially at the new minus ends. The timestamp refers to mm:ss. Acquisition rate: 5 fps, display rate: 24 fps. Related to Fig. 7 C.

These data show that freshly generated microtubule minus ends either obtained by laser-mediated severing or enzymatic release of γTuRC are recognized by NuMA.

### Full-length NuMA can mediate dynein/dynactin-driven microtubule transport

We have demonstrated that full-length NuMA activates dynein motility via its N-terminal part and binds to and stabilizes free microtubule minus ends via its C-terminal part. This raises the question of whether dynein together with full-length NuMA and dynactin can transport a microtubule minus end toward the minus end of another microtubule.

**Figure 8.**
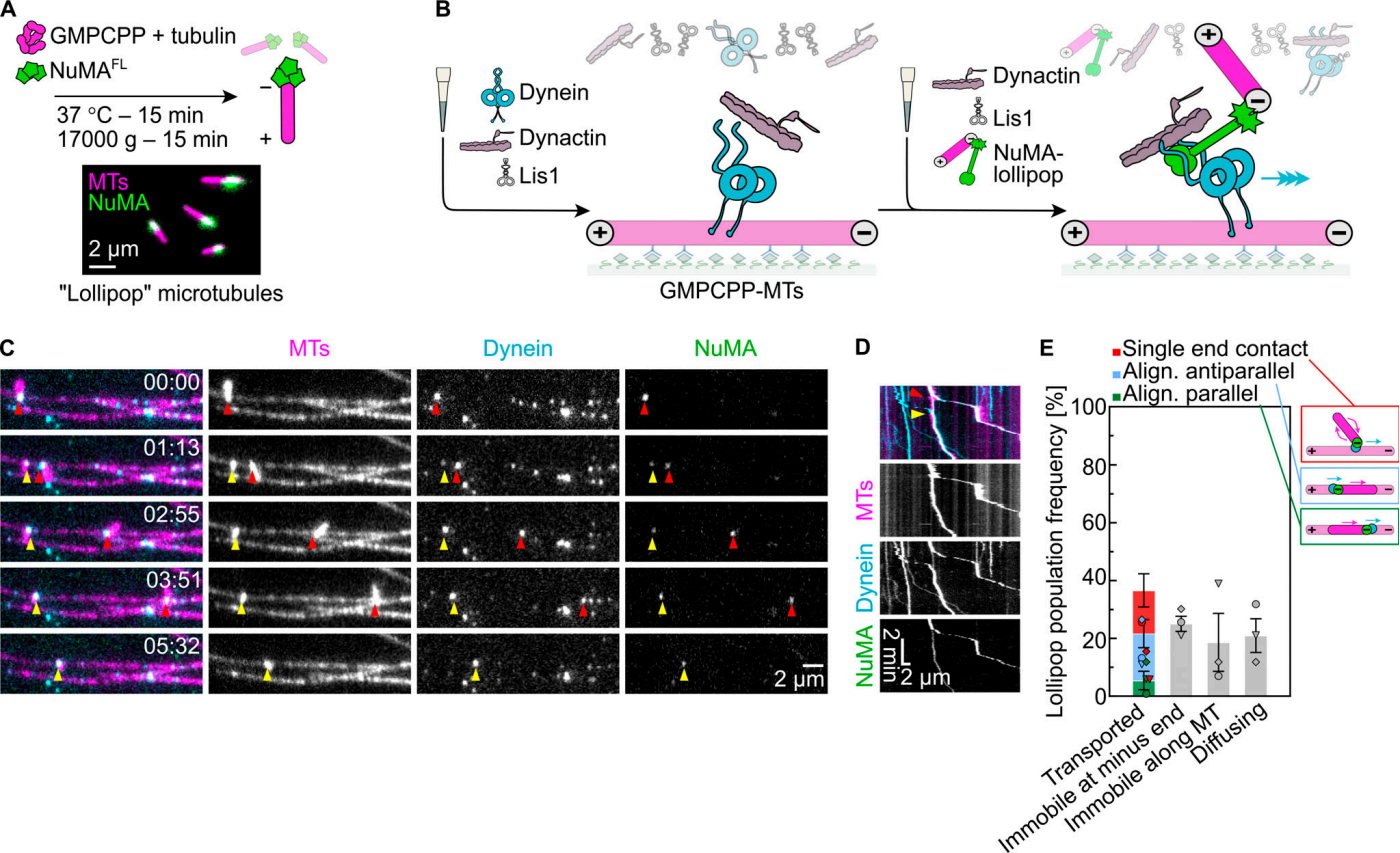
**Full-length NuMA can mediate dynein/dynactin-driven microtubule transport. (A)** Schematic (top) of the polymerization protocol to obtain microtubule “lollipops” with representative TIRF microscopy image (bottom) of lollipops obtained from a mixture of 0.8 µM Atto647N-tubulin (magenta), 40 nM mScarlet-NuMA^FL^ (green), and 1 mM GMPCPP. Minus ends can be identified by the selective presence of mScarlet-NuMA^FL^. **(B)** Schematic of a two-flush TIRF microscopy dynein-driven microtubule transport assay: first, dynein, dynactin, and Lis1 are flowed into a channel containing long surface-immobilized GMPCPP-microtubules; dynein is allowed to accumulate on microtubules for ≈3 min; in a second step, lollipops are introduced into the channel, with dynactin and Lis1, leading to dynein/dynactin/lollipop-bound NuMA transporting lollipop microtubules. **(C and D)** Representative time course TIRF microscopy images (C) and related kymograph (D) of transport events performed in the presence of 14 nM mEGFP-dynein (pre-bound to immobilized GMPCPP-microtubules), 28 nM dynactin and 1,000 nM Lis1. For both the faster (red arrowheads) and slower (yellow arrowheads) transport events, the lollipop is bound to the immobilized microtubule through a single anchoring point, corresponding to co-localizing mEGFP-dynein and mScarlet-NuMA^FL^ (arrowheads), resulting in it to dangle while being transported. Related to Video 4. **(E)** Left: frequency of lollipop microtubule behaviors on immobilized microtubules (mean ± SEM, three biological replicates); each distinct symbol represents a replicate; protein concentrations as in C and D; *n* = 82 landed lollipops. Right: cartoons representing the three categories of transported lollipops; dynein and NuMA are depicted in cyan and green, respectively, the lollipop microtubule is shorter and brighter; the polarity of both lollipop and immobilized microtubule are indicated; arrow points to the direction of dynein motility (blue) and microtubule transport (magenta). Experiments were performed in dynein microscopy buffer with the omission of methylcellulose.

**Figure S5.**
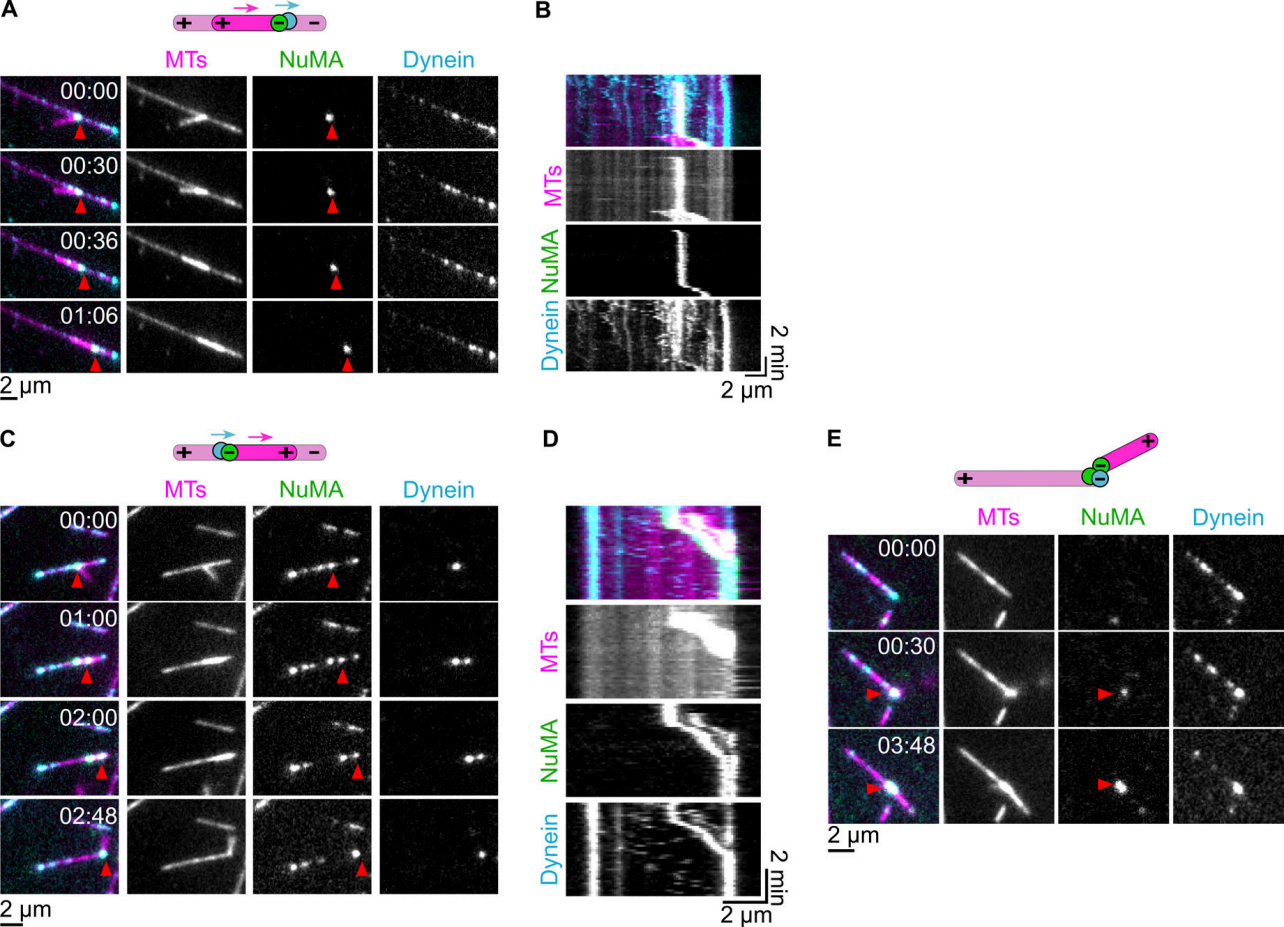
**Different modes of dynein/dynactin/NuMA** ^**FL**^ **-mediated microtubule transport. (A–E)** Representative time course TIRF microscopy images with schematics illustrating the type of transport (A, C, and E) and related kymographs (B and D) of microtubule transport experiments performed in the presence of 14 nM mEGFP-dynein (prebound to surface-immobilized Atto647N-labeled GMPCPP-microtubules), 28 nM dynactin, 1,000 nM Lis1, and “lollipop” microtubule minus-end bound mScarlet-NuMA^FL^. **(A and B)** A lollipop gets aligned to the immobilized microtubule, apparently through some crosslinking dynein bound at a distance from the lollipop minus end (red arrowhead, strongest NuMA signal). The orientation is parallel, as the lollipop minus end is facing the immobilized microtubule minus end (recognized by the direction of dynein-driven transport, and accumulated dynein). **(C and D)** Similarly to A and B, a lollipop is aligned to the immobilized microtubule, however, in antiparallel orientation, which results in the plus end of the lollipop being slid toward the minus end of the immobilized microtubule. **(E)** A lollipop-bound NuMA (red arrowhead) in solution lands directly on the minus end of the immobilized microtubule, where dynein has accumulated, resulting in transport-independent gathering of minus ends. The timestamps refer to mm:ss. Schematics as described in Fig. 8.

To test this, we allowed microtubules to nucleate and grow in suspension in the presence of NuMA^FL^ and GMPCPP, generating short stabilized microtubules with a strong accumulation of NuMA^FL^ at their minus ends (NuMA “lollipops”) (Fig. 8 A). These NuMA lollipops were then added to surface-immobilized long GMPCPP-microtubules that had been preincubated with dynein, dynactin, and Lis1 (Fig. 8 B). Lollipops landed sometimes with their NuMA-decorated minus ends on the immobilized microtubules, showing then different types of behavior: ≈37% of lollipops could be observed to be transported processively toward the minus end of the immobilized microtubule, with NuMA and dynein bound to the lollipop minus end (Fig. 8, C–E). About half of these transport events showed a “dangling” lollipop with its minus end bound via dynein and NuMA to the immobilized microtubule (Fig. 8, C–E and Video 4). In a minority of cases, the lollipop aligned in a parallel fashion to the immobilized microtubule, apparently forming additional crosslinks, in addition to the dynein/NuMA link at the minus end of the lollipop microtubule (Fig. 8 E; and Fig. S5, A and B). Unexpectedly, a considerable fraction of transported lollipops was aligned to the immobilized microtubule in an antiparallel fashion, with their minus ends facing the plus end of the immobilized microtubule, again indicating the presence of additional links along the length of the microtubules (Fig. 8 E; and Fig. S5, C and D).

**Video 4.** **Full-length NuMA can mediate dynein/dynactin-driven microtubule transport.** Microtubule transport events observed in the presence of 14 nM mEGFP-dynein (pre-bound to surface-immobilized Atto647N-labeled GMPCPP-microtubules), 28 nM dynactin, and 1,000 nM Lis1. Microtubule “lollipops” (bright magenta) landed from the solution are bound to the immobilized microtubule (dim magenta) through an anchoring point, corresponding to co-localizing mEGFP dynein (cyan) and mScarlet-NuMA^FL^ (green). This results in the lollipops to dangle while being transported processively, at different speeds, toward the minus end of the immobilized microtubule. The timestamp refers to mm:ss. Acquisition rate: 3.5 fps, display rate: 35 fps. Related to Fig. 8, C and D.

≈25% of the lollipops were bound in a static manner with their minus ends at the minus end of the immobilized microtubules (Fig. 8 E), a configuration that might have resulted from transport before imaging started. Alternatively, lollipops might have landed directly on the dynein-decorated minus ends of the immobilized microtubules (Fig. S5 E).

Lollipop microtubule transport velocities were varied, ranging from 3 to 400 nm s^−1^, considerably slower than the velocities of the dynein/dynactin/NuMA^FL^ complex alone (Fig. S2 B). This may be due to friction generated by some immobile dynein molecules that are typically observed under such in vitro conditions or by NuMA clusters that may generate immobile crosslinks between microtubules.

Taken together, these observations demonstrate that NuMA is a dynein adaptor that can bind microtubule minus ends as a cargo, thus enabling the dynein-driven gathering of minus ends.

## Discussion

Using in vitro reconstitution experiments, we established NuMA as a mitotic activating dynein adaptor and extended the list of categorized dynein cargoes, now including also microtubule minus ends. Our results support a model for spindle pole focusing where NuMA activates dynein/dynactin via its N-terminus and at the same time connects it to microtubule minus ends via its C-terminus, allowing minus ends to be transported to other microtubule minus ends.

We found that NuMA’s N-terminal part is sufficient to act as an activating dynein adaptor, in agreement with a recent report (Aslan et al., 2024, *Preprint*). NuMA contains both a Hook domain and a CC1-box-like motif (Renna et al., 2020), a unique combination among established dynein adaptors (Olenick and Holzbaur, 2019). In contrast to the adaptors BicD2N, Hook3, Ninein (Nin), Ninein-like (Ninl), BicDR1, and TRAK1 (McKenney et al., 2014; Schlager et al., 2014; Schroeder and Vale, 2016; Redwine et al., 2017; Urnavicius et al., 2018; Canty et al., 2023), efficient initiation of dynein’s processive motility by NuMA in the presence of dynactin is strictly dependent on the additional presence of Lis1, similar to TRAK2 and Hook2 (Christensen et al., 2021; Fenton et al., 2021; Canty et al., 2023). This dependence on Lis1 in our motility experiments with purified proteins agrees well with studies in cells and *Xenopus* egg extract showing that dynein, dynactin, NuMA, and Lis1 are all needed for correct spindle pole focusing (Wang et al., 2013; Monda and Cheeseman, 2018; So et al., 2022).

Whereas some dynein adaptors, such as BicDR1 and Hook3, appear not to be autoinhibited (Urnavicius et al., 2018; Kendrick et al., 2019) others like BicD1/2, Spindly, and JNK-interacting protein 3 (JIP3) can be autoinhibited (Liu et al., 2013; Carter et al., 2016; D’amico et al., 2022; Singh et al., 2023, *Preprint*). Full-length NuMA was able to promote processive dynein motility, indicating that under our conditions it is not or at least not completely autoinhibited. A meaningful quantitative comparison of the dynein activation efficiency of full-length NuMA and the N-terminal NuMA fragment was not possible due to the considerably different solubility of the two constructs under our motility assay conditions. We noted that a recent study suggests that mitotic kinases can activate NuMA (Aslan et al., 2024, *Preprint*). Future work will be required to establish to which extent NuMA may be autoinhibited or not.

Going beyond the previously known microtubule-binding capacity of NuMA’s C-terminal part, we discovered here that NuMA binds directly and preferentially to microtubule minus ends, stopping their growth and protecting them against shrinkage, effectively capping them, confirmed also by a recent study (Aslan et al., 2024, *Preprint*). The observation that γTuRC hinders NuMA’s minus-end binding may suggest that NuMA recognizes either the exposed α-tubulins at minus ends or even the interior of microtubules at their minus ends, both being made inaccessible by γTuRC (Aher et al., 2024; Brito et al., 2024; Dendooven et al., 2024). Structural studies will be needed to provide more insight into the mechanism of microtubule minus-end capping by NuMA.

The importance of both the N-terminal and C-terminal part being required for NuMA’s function to contribute to pole focusing is in agreement with experiments in cells or *Xenopus* egg extract showing that depleting NuMA (Merdes et al., 1996, 2000; Hueschen et al., 2017; Sun et al., 2021; van Toorn et al., 2022), removing, overexpressing, or mutating its N-terminal part (Kotak et al., 2012; Okumura et al., 2018; Renna et al., 2020), or removing its C-terminal part (Silk et al., 2009; Hueschen et al., 2017; Okumura et al., 2018) leads to strong spindle pole focusing defects. However, some reported results appear to contradict our simple model, especially when only parts of NuMA’s tail were removed, potentially suggesting that other NuMA interactors or posttranslational modifications not present in our reconstitutions may contribute to NuMA’s pole focusing function (Hueschen et al., 2017; Okumura et al., 2018).

NuMA is the only activating dynein adaptor known to date that connects dynein to a cytoskeletal filament. Linking a microtubule-dependent motor to a microtubule minus end generates a new type of active microtubule crosslinker. Dynein/dynactin/NuMA is different from the plus-end directed motor kinesin-5 (KIF11 in human) or kinesin-12 (KIF15 in human) that are symmetric crosslinkers with dimeric motors at both ends of the molecule, allowing them to move along two microtubules simultaneously (Kashina et al., 1996; Kapitein et al., 2005; Drechsler and McAinsh, 2016). The minus-end directed kinesin-14 (HSET or KIFC1 in human) is an asymmetric crosslinker that links two microtubules using a dimeric motor connected to a diffusive microtubule-binding domain without any microtubule end specificity (Fink et al., 2009; Hentrich and Surrey, 2010). This design, however, causes kinesin-14 to become a poor pole-focusing motor in the presence of kinesin-5 (Henkin et al., 2022). Similar to kinesin-14, dynein/dynactin/NuMA is also an asymmetric microtubule crosslinker with one motor end and a non-motor microtubule-binding end, however, the latter binds microtubules with a strong preference for minus ends. This design appears to be specialized for efficient minus-end focusing, suggesting that different motor designs have evolved for distinct functions. In the future, it will be interesting to understand how this new type of crosslinker cooperates with other microtubule crosslinking motors of different designs to ensure correct bipolar spindle organization.

We observed that γTuRC at microtubule minus ends prevents NuMA minus-end accumulation. We attribute the also observed apparent mild co-localization of NuMA with a minority of γTuRC-nucleated minus ends as random surface binding of NuMA close to the minus end. We can however not completely exclude that in rare cases NuMA may weakly bind to potential lattice defects at the γTuRC-microtubule interface (Vermeulen et al., 2024). Therefore, at least in the vast majority of cases, γTuRC competes with NuMA binding, and we showed that NuMA accumulation at minus ends of γTuRC-nucleated microtubules requires γTuRC-uncapping. This suggests that also in cells minus ends need to be uncapped from γTuRC or microtubules need to be severed before NuMA can accumulate at free minus ends, which can then be transported to the spindle pole by dynein. This model agrees with the observed enrichment of microtubule severing enzymes on spindles, especially near the poles (Jiang et al., 2017; Jin et al., 2017), and with the observed NuMA accumulation at the minus ends of kinetochore fibers after laser ablation in cells (Hueschen et al., 2017). The requirement for γTuRC uncapping may prevent excessive concentration of γTuRC at spindle poles, given that it also needs to be available throughout the spindle for augmin/RanGTP-dependent branched microtubule nucleation (Uehara et al., 2009; Petry et al., 2013). On the other hand, when being concentrated at poles by dynein transport, NuMA’s reported clustering and condensation activity may then further stabilize spindle poles (Okumura et al., 2018; Sun et al., 2021).

NuMA is also known to bind the membrane-anchored protein LGN with its C-terminal tail, thereby tethering dynein to the cortex and allowing dynein to position the spindle correctly in the cell (Okumura et al., 2018; Pirovano et al., 2019). Phosphorylation by the mitotic kinases CDK1 (Compton and Luo, 1995; Kotak et al., 2013; Seldin et al., 2013), Aurora-A kinase (Gallini et al., 2016; Kotak et al., 2016), and Polo-like kinase 1 (Plk1) (Kettenbach et al., 2011; Sana et al., 2018) has been shown to control the relative distribution and turnover of NuMA at the spindle poles and the cell cortex. In the future, it will be important to understand how these phosphorylations may selectively affect NuMA’s interaction with either cortical binding sites versus microtubule minus ends at the spindle poles, or with dynein/dynactin or other potential binding partners to achieve the right balance of dynein/NuMA activity at different cellular locations and times during mitosis.

## Materials and methods

All reagents were purchased from Sigma-Aldrich unless otherwise stated. Chromatography columns were purchased from Cytiva.

### DNA constructs

All NuMA constructs were generated from a plasmid, containing the coding sequence for full-length human NuMA isoform 1 (NM_006185), and codon-optimized for *Spodoptera frugiperda* expression (generous gift of Marina Mapelli). All vectors shared the same pFastBac1 (Invitrogen) backbone. NuMA^N-term^ was N-terminally tagged with sequences encoding for a Strep-tag II, a tobacco etch virus (TEV) protease cleavage site, a SNAP-tag, and a PreScission protease cleavage site. All other NuMA constructs encoded for an mScarlet instead of a SNAP-tag and lacked the PreScission site. To generate NuMA^N-term^, two consecutive stop codons were inserted after the coding sequence for the first 705 aa of full-length NuMA. To create NuMA^C-term L^, NuMA^C-term S1^, and NuMA^C-term S2^, the coding sequences for aa 1560–2115, 1701–1981 and 2002–2105, respectively, were amplified from the full-length sequence. Constructs containing the C-terminal part of NuMA have D1560T, T1820A, H2115InsLE changes compared with the sequence in the database.

To produce a construct for the simultaneous expression in insect cells of all six subunits of the human cytoplasmic dynein 1 complex, the biGBac system was utilized (Weissmann et al., 2016). First, we amplified the sequence encoding for an N-terminal His_8_-tag followed by a ZZ-tag, two TEV protease cleavage sites, an mEGFP, and the dynein heavy chain (DHC) (*DYNC1H1*, NM_001376.4), from the vector pGFPdyn1 (Jha et al., 2017), and cloned it into the pBIG1a vector. Compared to the canonical EGFP sequence, our gene contains two changes: (1) M1insS, resulting from the cloning process; (2) L221K, previously shown to promote a monomeric state (Zacharias et al., 2002; Snapp et al., 2003). Secondly, each of the sequences encoding for the smaller subunits intermediate chain 2C (IC2C) (*DYNC1I2*, AF134477), light intermediate chain 2 (LIC2) (*DYNC1LI2*, NM_006141.2), Tctex1 (*DYNLT1*, NM_006519.2), roadblock 1 (Robl1) (*DYNLRB1*, NM_014183.3), and light chain 8-1 (LC8-1) (*DYNLL1*, NM_003746.2) was amplified from a pDyn2 vector (Schlager et al., 2014) (generous gift of Andrew Carter) and separately cloned into the pLIB vectors. The individual sequences were then amplified from their respective pLIB and cloned together into the pBIG1b vector. Eventually, all dynein subunits were joined into the pBIG2ab vector.

The coding sequence for full-length human Lis1 was amplified from the plasmid His_6_-TEV-mCherry-Lis1-pFastBac1 (Jha et al., 2017) and cloned into the vector pFastBacHT A (Invitrogen) to obtain a construct for insect cell expression of unlabeled Lis1 N-terminally tagged with an His_6_-tag and a TEV protease cleavage site.

To generate a construct for bacterial expression of BicD2N^1–400^, the coding sequence for the first 400 aa of human BicD2 was amplified from a cDNA (SC300552; Origene) by PCR and cloned into a pETZT2 plasmid, inserting N-terminally a His_6_-tag, a Z-tag, and a TEV cleavage site.

Lentiviral vectors for the expression of a fluorescently tagged and biotinylatable human γTuRC were generated as detailed previously (Consolati et al., 2020).

The pFastbac vector for insect cell expression of human Strep-tag II-KIF2A and the pETMZ vector for bacterial expression of human His_6_-Ztag-TEV-Spastin were generated as described previously (Henkin et al., 2023).

### Cell lines

*Escherichia coli* strains DH5α, BL21-CodonPlus (DE3)-RIPL, and MAX Efficiency DH10Bac (Gibco) were grown in Luria Bertani (LB) medium (CRG Protein Technologies Unit) in the presence of appropriate antibiotics. For expression of recombinant proteins in insect cells, the *S. frugiperda* strain Sf21 (source: EMBL) was grown in suspension at 27°C in Sf-900TM III SFM Serum Free Medium (Gibco). The baculovirus preparation was carried out according to the manufacturers protocol (Bac-to-Bac system; Life Technologies), and baculovirus-infected insect cells were frozen to generate stable viral stocks similarly to what was described previously (Wasilko et al., 2009). For recombinant biotinylated mBFP-γTuRC expression, HeLa-Kyoto cells (RRID:CVCL_1922) were cultured, infected, and harvested as detailed previously (Consolati et al., 2020).

### Purification and labeling of recombinant human mScarlet-NuMA^N-term^

Recombinant human NuMA^N-term^ was purified from a pellet of 1.6 liters of Sf21 cell culture (≈16 g) resuspended in lysis buffer (50 mM sodium phosphate buffer, 300 mM KCl, 0.5 mM adenosine-5′-triphosphate [ATP]_,_ and 5 mM 2-mercaptoethanol [2-ME], pH 7.4) supplemented with 50 U/ml Benzonase (Merck-Millipore) and protease inhibitors (cOmplete EDTA-free Protease Inhibitor Cocktail [Roche Applied Science]). The lysis was carried out using an Avestin EmulsiFlex-C5 homogenizer (two rounds). After clarification of the lysate by centrifugation (15,800 × *g*, 30 min, 4°C), the supernatant was loaded onto a 5 ml StrepTrap HP column. The column was washed with lysis buffer and the elution was carried out using lysis buffer supplemented with 2.5 mM D-desthiobiotin. The NuMA-containing fractions were pooled, concentrated (Amicon Ultra 4, 30 kDa MWCO), and the Strep-tag II was cleaved off by incubating with TEV protease (CRG Protein Technology Unit) for 2 h at 4°C, using a TEV-to-protein ratio of 1:15 (wt/wt). During or after cleavage, 1.5 ml fractions of the protein pool were incubated with threefold molar excess of SNAP-Surface Alexa Fluor 546 or SNAP-Surface Alexa Fluor 647 (New England Biolabs) overnight at 4°C. The labeled protein was concentrated (Amicon Ultra 15, 50 kDa MWCO) and ultracentrifuged (278,088 × *g*, 10 min, 4°C). The unreacted dye was removed by size exclusion chromatography using a Superose 6 Increase 10/300 GL column and lysis buffer. The NuMA-containing peak fractions were pooled and concentrated to ≈0.4 mg ml^−1^. The yield was ≈0.2 mg protein/g pellet and the labeling efficiency was 95–100%.

### Purification of recombinant human mScarlet-NuMA^FL^

Recombinant human mScarlet-NuMA^FL^ was purified from a pellet of 2.1 liters of Sf21 cell culture (≈15 g) resuspended in lysis buffer (50 mM sodium phosphate buffer, 150 mM KCl, 1 mM EDTA ethylenediaminetetraacetic acid [EDTA]_,_ 2 mM MgCl_2_, 0.5 mM ATP, 0.02% Brij-35 [Thermo Fisher Scientific], and 5 mM 2-ME, pH 8.0) supplemented with 50 U/ml Benzonase, 50 µg/ml DNAse I (Roche Applied Sciences) and protease inhibitors. Lysis was carried out using a douncer homogenizer (Wheaton, 50 tight strokes). The lysate was supplemented with 350 mM KCl and incubated for 10 min on ice. After clarification by centrifugation (256,631 × *g*, 45 min, 4°C), the supernatant was loaded onto a 5 ml StrepTrap HP column, equilibrated with binding buffer (lysis buffer supplemented with 350 mM KCl and protease inhibitors). The column was washed with wash buffer (lysis buffer supplemented with 350 mM KCl and 4.5 mM ATP), and the protein was eluted using a binding buffer supplemented with 10 mM D-desthiobiotin. The NuMA-containing fractions were pooled and concentrated (Amicon Ultra 4, 100 kDa MWCO [Merck Millipore]). The Strep-tag II was cleaved off by incubating with TEV protease for 2 h at 4°C using a TEV-to-protein ratio of 1:10 (wt/wt) and the digested protein was ultracentrifuged (278,088 × *g*, 10 min, 4°C). The protein was further purified by size exclusion chromatography using a Superose 6 Increase 10/300 GL column with size exclusion buffer (50 mM sodium phosphate buffer, 300 mM KCl, 0.2% Brij-35, 2 mM DTT [NZYtech], pH 8.0) supplemented with protease inhibitors. The NuMA-containing peak fractions were pooled and concentrated to ≈1.5 mg ml^−1^. The yield was ≈0.3 mg protein/g pellet.

### Purification of recombinant human mScarlet-NuMA^C-term L^

Recombinant human NuMA^C-term L^ was purified from a pellet of 1.4 liters of Sf21 cell culture (≈10 g) and resuspended in a lysis buffer (50 mM sodium phosphate buffer, 500 mM KCl, 0.5 mM ATP_,_ and 5 mM 2-ME, pH 7.8) supplemented with 120 U/ml Benzonase, 2 mM phenylmethylsulfonyl fluoride (PMSF), and protease inhibitors. The purification protocol resembled that of NuMA^N-term^ with the following differences: (1) the speed of the first centrifugation was 20,400 × *g*; (2) after a first wash with lysis buffer supplemented with protease inhibitors, the StrepTrap HP column was washed with lysis buffer supplemented by 500 mM KCl; (3) the NuMA-containing StrepTrap HP eluate fractions were not concentrated prior to cleavage by TEV. The final pool of NuMA-containing peak fractions was concentrated to ≈0.6 mg ml^−1^. The yield was ≈0.5 mg protein/g pellet.

### Purification of recombinant human mScarlet-NuMA^C-term S1^

Recombinant human NuMA^C-term S1^ was purified from a pellet of 1 liter of Sf21 cell culture (≈11 g) resuspended in lysis buffer (50 mM KH_2_PO_4_, 50 mM Na_2_HPO_4_ [Thermo Fisher Scientific], 800 mM KCl, 2 mM MgCl_2,_ 1 mM EDTA, and 2 mM 2-ME, pH 8.0) supplemented with 50 U/ml Benzonase and protease inhibitors. Lysis was carried out using an Avestin EmulsiFlex-C5 homogenizer (two rounds). After clarification of the lysate by centrifugation (30,000 × *g*, 30 min, 4°C), the supernatant was filtered through a Millex-HV sterile syringe filter unit PVDF (0.45 μm; Merck) and loaded onto a 5 ml StrepTrap HP column. The column was washed with lysis buffer and eluted with Strep elution buffer (50 mM KH_2_PO_4_, 50 mM Na_2_HPO_4_, 500 mM KCl, 2 mM MgCl_2,_ 1 mM EDTA, 2 mM 2-ME, and 2.5 mM D-desthiobiotin, pH 8.0). The NuMA-containing fractions were pooled and concentrated (Amicon Ultra 4, 30 kDa MWCO). The Strep-tag II was cleaved off by incubating with TEV protease overnight at 4°C using a TEV-to-protein ratio of 1:30 (wt/wt). The cleaved protein was concentrated (Amicon Ultra 4, 30 kDa MWCO), ultracentrifuged (529,484 × *g*, 10 min, 4°C), and loaded on a HiLoad 16/600 Superdex 200 prep grade column. The elution was performed using size exclusion buffer (BRB80 [80 mM Pipes, 1 mM MgCl_2,_ 1 mM ethylene glycol bis(β aminoethyl ether) N,N,N′,N′ tetraacetic acid [EGTA]], 500 mM KCl, and 5 mM 2-ME, pH 6.8). The NuMA-containing peak fractions were pooled, exchanged into storage buffer (BRB80, 50 mM KCl, 5 mM 2-ME, pH 6.8), and concentrated (Amicon Ultra 4, 30 kDa MWCO) to ≈15 mg ml^−1^. The yield was ≈1 mg protein/g pellet.

### Purification of recombinant human mScarlet-NuMA^C-term S2^

Recombinant human NuMA^C-term S2^ was purified from a pellet of 0.9 liters of Sf21 cell culture (≈4 g) using the same protocol described for NuMA^C-term S1^ with two modifications: (1) prior to TEV cleavage, the protein was exchanged into cleavage buffer (50 mM KH_2_PO_4_, 50 mM Na_2_HPO_4_, 200 mM KCl, 2 mM MgCl_2,_ 1 mM EDTA, and 2 mM 2-ME, pH 7.0) using a PD-10 desalting column; (2) the size exclusion elution was carried out in storage buffer, which avoided the final buffer exchange step. The protein was concentrated to ≈24 mg/ml and the yield was ≈4 mg protein/g pellet.

### Purification of recombinant human mEGFP-dynein complex

Recombinant human mEGFP-dynein was purified from a pellet of 1.4 liters of Sf21 cell culture (≈16 g) as described (Jha et al., 2017) with the following modifications: (1) 250 mM K-acetate in the lysis buffer was substituted by 150 mM KCl; (2) the lysate was clarified twice by ultracentrifugation (125,749 × *g*, 20 min, 4°C; 225,634 × *g*, 20 min, 4°C); (3) size exclusion chromatography was performed using a HiLoad 16/600 Superose 6 prep grade column with size exclusion buffer (50 mM HEPES, 400 mM K-acetate, 2 mM MgSO_4_, 0.1 mM ATP, and 5 mM DTT, pH 7.4). The dynein-containing peak fractions were concentrated (Amicon Ultra 4, 50 kDa MWCO) to ≈1.2 mg ml^−1^. The yield was ≈0.3 mg protein/g pellet.

### Purification of porcine brain dynactin

Endogenous brain dynactin was purified from two pig brains by BicD2N^1–400^ affinity chromatography followed by ion exchange chromatography, as described (Jha et al., 2017), with the following modifications: (1) to generate the BicD2N^1–400^ column, 60 mg of purified His_6_-tag-Z-tag-TEV-BicD2N^1–400^ (see below) were conjugated to a 5 ml HiTrap NHS-Activated HP column following the manufacturer’s recommended method, with a ≈90% yield; (2) two intermediate steps of washing with lysis buffer were carried out during the application of the lysate to the BicD2 column to avoid overloading and clogging; (3) the dynein–dynactin complex was eluted at 3 ml/min using lysis buffer supplemented with 1 M KCl through a 25 ml step gradient from 0 to 500 mM KCl; (4) the flow-through was collected and reloaded onto the BicD2N^1–400^ column to recover leftover unbound dynein–dynactin complex of the first loading, for a maximum of two times; (5) for the elution of the MonoQ 5/50 GL column, the following gradient was applied, using MonoQ binding buffer supplemented with 800 mM NaCl: linear 0–224 mM in 10 ml; step 224–256 mM in 1 ml; linear 256–272 mM in 20 ml; step 272–312 mM in 1 ml; linear 312–400 mM in 10 ml. The dynactin complex eluted at 330 mM NaCl. The dynactin-containing peak fractions were concentrated (Amicon Ultra 4, 50 kDa MWCO) to ≈0.6 mg ml^−1^. The yield was ≈75 µg protein/g brain.

### Purification of recombinant human Lis1 constructs

Recombinant human mCherry-Lis1 was purified according to a published protocol (Jha et al., 2017). Unlabeled Lis1 was purified from a pellet of 0.8 liters of Sf21 cell culture (≈11 g) resuspended in lysis buffer (50 mM sodium phosphate, 500 mM NaCl, 20 mM imidazole, 2 mM MgCl_2_, 10% glycerol [vol/vol, Thermo Fisher Scientific], 0.5 mM ATP, and 5 mM 2-ME, pH 7.4) supplemented with 20 µg/ml DNAse I, protease inhibitors, and 0.5% Triton X-100 (vol/vol). Cells were lysed using a douncer homogenizer (50 tight strokes). The lysate was clarified by centrifugation (104,350 × *g*, 15 min, 4°C) and run on a 5 ml HisTrap FF column. The column was washed first with lysis buffer supplemented with 500 mM NaCl, 4.5 mM ATP, and 8 mM MgCl_2_; and second with two-step gradients using lysis buffer supplemented with 330 mM imidazole: (1) 20–27.5 mM imidazole; (2) 27.5–55 mM imidazole. The protein was eluted using lysis buffer supplemented with 330 mM imidazole. The protein-containing fractions were pooled and exchanged into size-exclusion buffer (50 mM HEPES, 300 mM KCl, 0.05 mM ATP, 10% glycerol [vol/vol], 2 mM DTT, pH 7.4) using a HiTrap desalting column. The His_6_-tag was cleaved off by overnight incubation with TEV protease at 4°C using a TEV-to-protein ratio of 1:50 (wt/wt). The cleaved protein was concentrated (Amicon Ultra 15, 30 kDa MWCO), ultra-centrifuged (278,088 × *g*, 10 min, 4°C), and loaded onto a Superdex 200 10/300 GL column. The elution was performed with size-exclusion buffer; the Lis1-containing peak fractions were pooled and concentrated (Amicon Ultra 4, 30 kDa MWCO) to ≈9 mg ml^−1^. The yield was ≈0.3 mg protein/g pellet.

### Purification of recombinant human BicD2N^1–400^

Recombinant human BicD2N^1–400^ was expressed in *E. coli* BL21-CodonPlus (DE3)-RIPL cells by induction with 0.5 mM IPTG for 16 h at 16°C. A pellet of 1 liter of culture (≈4 g) was resuspended in lysis buffer (50 mM HEPES, 400 mM KCl, 2 mM MgCl_2_, 0.1 mM ATP, 10 mM imidazole, and 2 mM 2-ME, pH 7.4) supplemented with 2 mM PMSF and protease inhibitors. Lysis was carried out using an Avestin EmulsiFlex-C5 homogenizer (two rounds). The lysate was clarified by centrifugation (256,631 × *g*, 20 min, 4°C) and run over two 5-ml HisTrap HP columns. The columns were washed with wash buffer (50 mM HEPES, 1 M KCl, 2 mM MgCl_2_, 2 mM ATP, and 2 mM 2-ME, pH 7.4), and the elution was performed with lysis buffer supplemented with 490 mM imidazole. For each column, the flow-through was reloaded to recover the leftover unbound BicD2N^1–400^. The protein-containing fractions were pooled and the His_6_-tag was cleaved off by overnight incubation with TEV protease at 4°C using a TEV-to-protein ratio of 1:30 (wt/wt). The cleaved protein was concentrated (Amicon Ultra 15, 30 kDa MWCO), ultracentrifuged (529,484 × *g*, 10 min, 4°C, 15 min, 4°C), and loaded onto four HiLoad 16/600 Superdex 200 prep grade columns. The elution was carried out using size exclusion buffer (50 mM HEPES, 200 mM KCl, 1 mM MgCl_2_, 10% glycerol [vol/vol], and 1 mM 2-ME, pH 7.4). The pool of BicD2N^1–400^-containing peak fractions was split into two parts: the one destined for NHS-coupling (see dynactin purification above) was kept at ≈3.4 mg ml^−1^; the one destined for microscopy assays was concentrated (Amicon Ultra 4, 30 kDa MWCO) to ≈12 mg ml^−1^. The yield was ≈5 mg protein/g pellet.

### Purification of recombinant human biotinylated mBFP-γTuRC, human KIF2A, and human spastin

Recombinant human biotinylated mBFP-γTuRC was purified from HeLa-Kyoto cells as described previously (Brito et al., 2024). Recombinant human KIF2A was purified from Sf21 cells as described previously (Henkin et al., 2023). Recombinant human spastin was isolated from BL21 pRIL *E. coli* cultures as described previously (Henkin et al., 2023).

### Purification and labeling of porcine brain tubulin

Endogenous tubulin was isolated from the pig brain following sequential cycles of polymerization–depolymerization as previously described (Hyman, 1991; Castoldi and Popov, 2003). Tubulin was further purified by recycling and part of it was labeled with Atto647-NHS, Atto647N-NHS, and EZ-Link NHS-Biotin (Thermo Fisher Scientific), according to published methods (Consolati et al., 2022).

### Chromatography and protein concentrations

All purification steps were carried out at 4°C; all buffers were degassed and chilled to 4°C prior to pH adjustment; all types of chromatography were performed using an ÄKTA Pure System. At the final stage of every purification, the protein-containing peak fractions of the size exclusion eluate were identified by sodium dodecyl sulfate polyacrylamide gel electrophoresis (SDS-PAGE) Coomassie Blue G-250 staining. The concentrated fraction pool was ultracentrifuged (278,088 × *g*, 10 min, 4°C) and flash-frozen prior to long-term storage in liquid nitrogen. The protein concentrations were calculated after freeze–thawing from the absorbance measured at 595 nm via Bradford assay (Protein Assay Dye Reagent Concentrate [Bio-Rad]). For NuMA^C-term L^, the concentration was derived by SDS-PAGE Coomassie Blue G-250 staining quantification. For Atto647N-tubulin, Atto647-tubulin, AF546-NuMA^N-term^, and AF647-NuMA^N-term^, the dye concentration was obtained from the absorbance measured at the dye-specific wavelength and the extinction coefficient of the dye. Protein concentrations refer to monomers, except for tubulin concentrations, which refer to heterodimers, and dynein and dynactin concentrations refer to one copy of the entire complex.

### SDS-PAGE and western blotting

Protein samples were resolved by SDS-PAGE (Fig. 2 B; Fig. S1 A; and Fig. S3, A and B) using two electrophoresis systems: (1) XCell SureLock Mini-Cell (Invitrogen), in combination with NuPAGE Bis-Tris Mini protein gels (Invitrogen), NuPAGE LDS Sample Buffer 4X (Invitrogen), NuPAGE MES, and MOPS SDS running buffers 20X (Invitrogen); (2) Mini-PROTEAN Tetra Cell (Bio-Rad), in combination with Mini-PROTEAN TGX precast protein gels (Bio-Rad), Laemmli SDS sample buffer reducing 6X (Alfa Aesar), and XT-Tricine running buffer (Bio-Rad). Precision Plus Protein Dual Xtra (Bio-Rad) and HiMark (Invitrogen) were used as prestained protein standards. Gels were run according to the manufacturers’ recommendations. Gel staining was performed using InstantBlue Coomassie protein stain (Abcam) or Coomassie Brilliant Blue R-250 dye (Thermo Fisher Scientific).

For immunoblotting of dynein and dynactin subunits (Fig. S1 A), the gels were transferred to iBlot2 Transfer Stacks PVDF (0.2 µm; Invitrogen) using the iBlot 2 Gel Transfer Device (Invitrogen). Dynein heavy chain subunit was transferred at 25V for 12 min; dynein lighter chains using the default protocol “P3”; and all dynactin subunits using the default protocol “P0.” Membrane blocking and antibody dilution buffer consisted of tris-buffered Saline (TBS) or phosphate-buffered saline (PBS) (CRG Protein Technologies Unit), 5% skimmed milk powder (wt/vol; Millipore), and 0.05% Tween20 (vol/vol). Anti-GFP, anti-dynein antibodies, anti-dynactin antibodies, and HRP-conjugated secondary antibodies were diluted according to the manufacturers’ recommendations. Stained gel imaging and blot chemiluminescent detection were carried out using iBright CL1500 Imaging System (Invitrogen). Fluorescence visualization of mScarlet-tagged NuMA bands (Fig. S3 B) was performed using Molecular Imager Gel Doc XR System (Bio-Rad).

### Antibodies

The following commercial antibodies were utilized to perform the western Blots shown in Fig. S1 A:

Anti ACTR1A/dynactin Arp1 (PA5 30356; Invitrogen)
Anti CAPZB/dynactin CapZβ (A304 734A M; Bethyl Laboratories)
Anti DCTN2/dynactin p50 (A303-488A; Bethyl Laboratories)
Anti DCTN4/dynactin p62 (A304-986A-T; Bethyl Laboratories)
Anti DYNLL1/dynein LC8 (ab51603; Abcam)
Anti DYNLRB1/dynein Roadblock1 (STJ117391; St John’s Laboratory)
Anti DYNLT1/dynein TcTex1 (11954-1-AP; Proteintech)
Anti p150 [Glued] (610474; BD Biosciences)
Anti Rabbit Immunoglobulins/HRP (P0399; Agilent).

### Mass photometry

The oligomerization state of all proteins introduced in this study, except for mScarlet-NuMA^FL^, was analyzed using a TwoMP mass photometer (Refeyn) (Fig. S1 B and Fig. S3 C). Uncoated, clean Sample Carrier slides (Refeyn) and 6-well sample cassettes (Refeyn) were used for sample loading. All measurements and dilutions were executed with the respective TIRF microscopy assay buffer; methylcellulose was however excluded from the buffer. For each sample, 18 µl of buffer was loaded onto a well (3 mm diameter; Refeyn) for the autofocus; 2 µl of protein dilution was then mixed into the buffer drop to reach a final concentration of 5–20 nM. Protein contrast count was acquired with at least two technical replicates. The data acquisition time for each sample was 60 s. Molecular weights were assigned by comparison with calibration probes of known mass (Native Mark unstained protein standard [Invitrogen], β-amylase). All data were processed using the DiscoverMP software (Refeyn).

### Stabilized microtubules

#### Stabilized microtubules for microtubule sedimentation assays

Microtubules for sedimentation assays were prepared from a mixture containing 15 µM tubulin, including 16% (mol/mol) of Atto647N-tubulin (for a final fluorescent labeling ratio of 6.2%); 1 mM guanosine 5’ [(α,β) methyleno] triphosphate (GMPCPP) (Jena Bioscience); and BRB80 to reach a final volume of 60 µl. The mixture was incubated for 5 min on ice and then 1 h at 37°C. The microtubules were centrifuged in a tabletop centrifuge at 17,000 × *g* for 20 min; the pellet was resuspended in 100 µl BRB80T (BRB80 supplemented by 10 µM Paclitaxel), re-centrifuged at 17,000 × *g* for 10 min, finally resuspended in the same volume of BRB80T, and used within the same day.

#### Long stabilized microtubules for microscopy

To generate stabilized long microtubules, we incubated 1.3 µM tubulin containing 43% (mol/mol) biotin-labeled tubulin and 10% (mol/mol) of Atto647N-tubulin (for a final fluorescent labeling ratio of 3.8%) in BRB80 with 1 mM GMPCPP, 1.25 mM MgCl_2_, and 1 mM tris(2 carboxyethyl)phosphine (TCEP) (90 µl final volume) for 5 min on ice, and then at 27°C overnight. Centrifugation was carried out as described above for stabilized microtubules for microtubule sedimentation assays, and the pellet was resuspended in 90 µl BRB80T. These long, biotin and Atto647N-labeled microtubules were stored at room temperature and used for up to 2 wk. Prior to every usage, they were centrifuged as described above. For microscopy assays, the microtubule preparation was diluted up to fivefold in BRB80T.

#### Short stabilized microtubules (seeds) for microscopy

Stabilized short and bright GMPCPP-microtubules were polymerized as described for long ones, with the following differences: (1) the tubulin concentration in the polymerization solution was increased to 3 µM; (2) the tubulin mixture included 33% (mol/mol) Atto647N-tubulin (for a final fluorescent labeling ratio of 13%); (3) the final volume was 70 µl; (4) polymerization was performed at 37°C for 1 h; (5) resuspension after centrifugation was carried out in BRB80. These short, biotin and brightly Atto647N-labeled microtubules were either used on the same day or, for later use, they were supplemented with 10% glycerol (vol/vol), aliquoted, snap-frozen, and stored in liquid nitrogen. Short microtubules were diluted up to 200-fold in BRB80 before microscopy experiments.

### Microtubule sedimentation assay

1 µM of test protein was mixed with stabilized microtubules for microtubule sedimentation assays (0.5 µM polymerized tubulin) in NuMA microscopy buffer (BRB80, 50 mM KCl, 1 mM MgCl_2_, 1 mM EGTA, 2 mM guanosine-5′-triphosphate [GTP] [Jena Bioscience], 0.15% [wt/vol] methylcellulose, 1% [wt/vol] glucose, 1 mM TCEP). The appropriate amount of KCl was supplemented to achieve a final concentration of 60 mM KCl, accounting for the KCl carried by the various NuMA constructs from their respective storage buffers. The final reaction volume was 30 µl. The mixture was incubated at 30°C for 15 min. For negative controls, proteins were incubated in the same buffer without microtubules, and microtubules were incubated without proteins. Reaction mixtures were centrifuged in a tabletop centrifuge at 17,000 × *g* for 20 min; 20 µl of supernatant was removed from the top, while the bottom fraction in contact with the pellet was discarded. Pellets were resuspended in 30 µl using NuMA microscopy buffer. Samples were separated by SDS-PAGE and stained using InstantBlue Coomassie protein stain.

### Total internal reflection fluorescence (TIRF) microscopy

#### Flow chambers for TIRF microscopy assays

##### Chambers with surface-immobilized long or short microtubules

For assays with surface-immobilized GMPCPP-microtubules, glass coverslips (Menzel coverslips #1.5 18 × 18 and 22 × 22 mm; Epredia) were silanized (hexamethyldisilazane [HDMS]) according to a published protocol (Wedler et al., 2022) using HCl (Thermo Fisher Scientific) for activation. Chambers were assembled with these silanized, hydrophobic coverslips as described (Gell et al., 2010) using parafilm strips as spacers between two coverslips to create flow channels (18 mm long, ≈3 mm wide, ≈0.1 mm thick).

To immobilize biotinylated GMPCPP-microtubules via NeutrAvidin (Invitrogen), the following sequence of solutions was flowed through the channels at room temperature: (1) TetraSpeck microspheres 0.2 μm (Invitrogen) (4.6 × 10^9^ particles/ml in BRB80, added to allow for channel alignment and drift correction in postprocessing of recorded movies), incubated for 2 min; (2) BRB80, twice; (3) 0.4 mg/ml of NeutrAvidin in BRB80, incubated for 5 min; (4) BRB80; 5% Pluronic F-127 in BRB80, incubated for 10 min; (5) BRB80, twice; (6) biotinylated Atto647N-labeled GMPCPP-microtubules, incubated for up to 5 min (depending on the concentration of the stock); and (7) assay buffer. For all solutions, the volume was 15 µl, except for microtubules which were suspended in 5 µl. TetraSpeck microspheres and NeutrAvidin dilutions were freshly prepared on the day of the experiment and stored on ice.

##### Chambers with surface-immobilized γTuRC

For γTuRC nucleation assays, flow chambers were assembled with biotin-polyethylene glycol-functionalized glass as described previously (Consolati et al., 2022). Channel flowing and γTuRC immobilization was performed as described (Brito et al., 2024).

#### TIRF microscopy assays

##### Dynein motility assays

A dynein/dynactin premix of 10 µl was prepared by diluting dynein and dynactin stocks to a molar ratio of 1:2 (dynein complex: dynactin complex) in dynein microscopy buffer (BRB20 [20 mM Pipes, 1 mM MgCl_2_, 1 mM EGTA, pH 6.8], supplemented with 1 mM MgCl_2_, 2.5 mM ATP, 0.15% [wt/vol] methylcellulose, 1% glucose [wt/vol], 1 mM TCEP) to a dynein/dynactin concentration approximately sixfold higher than the final concentration. AF546-NuMA^N-term^ and AF647-NuMA^N-term^ stocks were diluted in dynein microscopy buffer; and NuMA^FL^, Lis1, and BicD2N^1–400^ stocks were diluted in BRB20 supplemented by 100 mM KCl and 2 mM TCEP.

For each condition, the appropriate volumes of diluted NuMA, Lis1, and BicD2N^1–400^ were added to 1.25 or 2.5 µl of dynein–dynactin premix (for Fig. 1, I–K and Fig. S2 B [NuMA experiments], Fig. S2 D or for all the other assays, respectively) to achieve the desired final concentrations of 3–7 nM dynein, 7–14 nM dynactin, 50–500 nM AF546-NuMA^N-term^, 50 nM AF647-NuMA^N-term^, 50 nM NuMA^FL^, 10–5,000 nM mCherry-Lis1, 650 nM Lis1 (as stated in the Fig. 1 and Fig. S2) in a total volume of 15 µl. The final volume was reached by adding dynein microscopy buffer, supplemented with oxygen scavenger mix (150 µg/ml catalase, 625 µg/ml glucose oxidase [SERVA Electrophoresis]) and KCl. The appropriate amount of KCl to reach a final concentration of 40 mM was adjusted for each condition considering the amount of KCl contributed by NuMA, Lis1, and BicD2N^1–400^ from their respective storage and dilution buffers. For control experiments in the absence of Lis1 or NuMA, their respective dilution buffer was added instead of the protein to maintain the same buffer composition. The final assay mix (15 µl) was flushed all at once in a channel containing immobilized long microtubules at 30°C in the TIRF microscope incubator (OkoLab).

##### NuMA binding to surface-immobilized GMPCPP-seeds and microtubules elongating from surface-immobilized GMPCPP-seeds

NuMA^FL^, NuMA^C-term L^, NuMA^C-term S1^, and NuMA^C-term S2^ premixes were prepared by diluting stock proteins to a concentration 10-fold higher than the final desired concentrations using BRB80, supplemented with 2 mM TCEP and the appropriate amount of KCl to achieve a final concentration of 100 mM KCl, accounting for the KCl carried by the various NuMA constructs from their respective storage buffers. A 150 µM tubulin premix, including 6% (mol/mol) of Atto647N-tubulin (corresponding to a final fluorescent labeling ratio of 2.4%), was prepared in BRB80.

NuMA premixes and the tubulin premix were diluted 10-fold and 15-fold, respectively, into a final volume of 30 µl with NuMA microscopy buffer (see Microtubule sedimentation assays), supplemented with oxygen scavenger mix, to reach the final concentrations of 25–40 nM NuMA^FL^, 10–75 nM NuMA^C-term L^, 40–1,000 nM NuMA^C-term S1^, 25–75 nM NuMA^C-term S2^, and 10 µM tubulin (as stated in Fig. 2, Fig. 3, Fig. 4, and Fig. S4). For control experiments in the absence of NuMA or tubulin, the respective dilution buffer was added instead of the protein to maintain the same buffer composition. The final assay mix (30 µl) was flowed in the microscopy channel in two steps of 15 μl, with a waiting time of 1 min between flows, at 30°C in the TIRF microscope incubator (for channels with immobilized short stabilized microtubules) or at room temperature (for channels with microtubules elongating from immobilized seeds). For experiments involving “pre-elongated” microtubules (Fig. 4, Fig. S4 C, and Video 1), the initial flush was performed at RT (tubulin only mix), while the second flush (NuMA and tubulin mix) was executed at 30°C in the TIRF microscope while imaging after the first ≈10 min of imaging.

##### NuMA binding to microtubules nucleated by surface-immobilized γTuRC

NuMA^FL^ premixes were prepared by diluting the stock protein into BRB80 supplemented with 2 mM TCEP and diluted to a concentration 10-fold higher than the final desired concentration.

NuMA^FL^ premixes were diluted 10-fold into a final volume of 80 µl with γTuRC microscopy buffer (BRB80, 60 mM KCl, 1 mM GTP, 5 mM 2-ME, 0.15% [wt/vol] methylcellulose, 1% [wt/vol] glucose, and 0.02% [vol/vol] Brij-35) supplemented with oxygen scavengers (0.1 mg/ml catalase, 1 mg/ml glucose oxidase) to reach the final concentrations of 5–30 nM NuMA^FL^, as indicated in Fig. 5. In control experiments without NuMA, an equivalent volume of NuMA^FL^ dilution buffer was used. A tubulin mix, including 38% (mol/mol) of Atto647-tubulin (for a final fluorescent labeling ratio of 5%), was added to the final mix upon the addition of NuMA^FL^ to reach a final concentration of 10 µM. The final assay mix was centrifuged in a 4°C tabletop centrifuge at 17,000 × g for 5 min, the supernatant was recovered, returned to a tube on ice, and flowed (60 µl) in the channel containing immobilized γTuRC at room temperature. Flowing was executed in two steps of 30 μl, with a waiting time of 1 min between flows.

##### NuMA binding to γTuRC-released microtubules

The γTuRC release assays presented in Fig. 6 were performed as described in NuMA binding to microtubules nucleated by surface-immobilized γTuRC, with the addition of KIF2A and spastin and small modifications to the buffer. KIF2A and spastin premixes were obtained by diluting the stock proteins into KIF2A gel filtration buffer (50 mM Na-phosphate, 300 mM KCl, 1 mM MgCl_2_, 1 mM EGTA, 5 mM 2-ME, 0.1 mM ATP, pH 7.5). Both proteins were diluted to a concentration 10-fold higher than the final desired concentration.

NuMA^FL^, KIF2A, and spastin premixes were diluted 10-fold into a final volume of 80 µl with γTuRC release buffer (γTuRC microscopy buffer supplemented with 5 mM Na-phosphate, 1.1 mM EGTA, 2 mM MgCl_2_, 1 mM ATP) to reach the final concentrations of 30 nM NuMA^FL^, 20 nM KIF2A, and 10 nM spastin.

##### NuMA binding to laser-ablated microtubules

NuMA^FL^ and tubulin premixes were diluted and added to the final assay mix as explained in NuMA binding to surface-immobilized seeds and microtubules elongating from surface-immobilized seeds to reach the final concentrations of 40 nM and 10 µM, respectively. The final assay mix (30 µl) was flowed in a channel containing immobilized seeds in two steps of 15 μl, with a waiting time of 1 min between flows, at 30°C in the TIRF microscope incubator.

After flushing the final assay mix into the channel and starting the imaging, NuMA was allowed to accumulate on the minus end of the GMPCPP-microtubules, while the plus end would start to elongate, for ≈3 min. This facilitated distinguishing the two ends. Subsequently, microtubule laser ablation was performed and, immediately afterwards a fresh assay mix was flushed once again into the channel while imaging.

##### Microtubule transport by dynein/dynactin/NuMA

Microtubules with NuMA-decorated minus ends in suspension (lollipop microtubules) were obtained using the following polymerization mixture: 0.8 µM tubulin, including 33% (mol/mol) of Atto647N-tubulin (for a final fluorescent labeling ratio of 13%); 40 nM NuMA^FL^; 1 mM GMPCPP; 1.4 mM MgCl_2_; 8 mM DTT; and BRB80 to reach a final volume of 35 µl. The mixture was incubated for 5 min on ice and then 20 min at 37°C. Microtubules were centrifuged in a tabletop centrifuge at 17,000 × *g* for 10 min and the pellet was resuspended in 7 µl of BRB80 supplemented with 10 mM DTT. Lollipops were stored at room temperature and used within an hour.

The dynein/dynactin premix was prepared as described in the dynein motility assay; Lis1 premix was prepared by diluting the stock protein 20-fold in BRB20 supplemented by 100 mM KCl and 2 mM TCEP.

Final mix A was prepared as follows: 1.6 µl of Lis1 premix was added to 5 µl of dynein/dynactin premix; the dynein/dynactin/Lis1 concentrated solution was diluted into dynein microscopy buffer to reach the final concentrations of 14 nM dynein, 28 nM dynactin, and 1,000 nM Lis1 in a final volume of 15 µl. Methylcellulose was omitted from the composition of the dynein microscopy buffer to avoid crowding-induced surface localization of lollipop microtubules. The assay buffer was supplemented with oxygen scavenger mix and KCl, adjusted as explained in the dynein motility assay. Final mix A (15 µl) was flowed into a channel containing surface-immobilized long microtubules at 30°C in the TIRF microscope incubator and incubated for up to 5 min to allow accumulation of dynein on microtubules.

During the incubation, final mix B was prepared as described for final mix A, leaving out dynein and introducing up to 4 µl of lollipop microtubules, which were added prior to warming up mix B tube at room temperature.

Assay premixes and final mixes were prepared on ice. BRB80 and BRB20 were prepared as 1× stocks, aliquoted, and kept at −20°C for long-term storage; once defrosted, they were stored at 4°C for usage up to 2 wk. Microscopy buffers were freshly prepared for the day of the experiment and stored on ice. After flushing the final assay mix, channels were sealed using vacuum grease (Dow Corning), immediately followed by TIRF microscopy imaging. For the assays which required multiple flushes, the channels were sealed after the last flush.

#### TIRF microscopy imaging

For TIRF images shown in Fig. 3, B, C, and E (75 nM kymograph) and Fig. 3 G; Fig. 4, C and D; Fig. 5; Fig. 8; Fig. S4 A; and Videos 2 and 4, TIRF microscopy was performed using an automated Nikon Eclipse Ti-E with Perfect Focus System, a 100× oil immersion TIRF objective (NA = 1.49, CFI SR Apo; Nikon), 1.3× additional magnification, and Andor iXon 888 Ultra EMCCD cameras (pixel size = 100 nm; Andor Technology) and controlled by MetaMorph software (Molecular Devices). The sample was excited using 360° TIRF illumination (iLas2; Gataca Systems). The following filter combinations were used: a 405 nm TIRF filter set (TRF49901; Chroma) with an additional ET460/50 (Chroma) bandpass filter; a 488 nm TIRF filter set (TRF49904; Chroma) with an additional ET525/50 (Chroma) bandpass filter; a 561 nm TIRF filter set (TRF49909; Chroma) with additional ET607/70 (Chroma) bandpass filter; a 638 nm TIRF filter set (TRF49914; Chroma) with additional LP655 (Chroma) long-pass filter.

For the TIRF images shown in all other figures (except Fig. 1, I and K), Videos 1 and 3, the imaging was executed on a similar TIRF setup, which differed only for the following components: (1) Nikon Eclipse Ti2-E with Perfect Focus System, (2) Andor iXon 897 Ultra EMCCD cameras (pixel size = 159 nm; Andor Technology); (3) iLas3 laser illumination (Gataca Systems).

For the NuMA/dynein co-localization experiments shown in Fig. 1, I and K, microscopy was carried out using an iMIC (TILL Photonics) TIRF microscope equipped with: a 100× oil immersion objective lens (NA = 1.49; Olympus); 1.26× additional magnification; Evolve 512 EMCCD cameras (pixel size = 127 nm; Photometrics); and a quadband filter (405/488/561/640; Semrock). The sample was excited via 360° TIRF illumination, and the system was controlled by Live Analysis software (TILL Photonics).

Typically, snapshots and time-lapse images were acquired with 100 ms exposure for all excited channels (mBFP-γTuRC: 405 nm; mScarlet-NuMA^FL^ and C-terminal constructs: 561 nm; Atto647-tubulin and Atto647N-tubulin: 638 nm) via sequential dual or triple-color imaging. Time-lapses were imaged using an acquisition rate of either: 2 fps (Fig. 6 and Video 2), between 3.5 and 6 fps (Fig. 8, Fig. S5, and Video 4), 5 fps (Fig. 7 and Video 3), 10 fps (Fig. 5), or between 5 and 15 fps (Fig. 3, Fig. 4, Fig. S4, and Video 1). Recording duration was 20–30 min. For the assays displayed in Fig. 1, C, F, and J; and Fig. S2 A, mEGFP-dynein imaging (488 nm excitation) was obtained through stream acquisitions of 2,000 frames with 50 ms of exposure. For the NuMA/dynein co-localizations shown in Fig. 1, I and K, visualization was performed by simultaneous dual-color imaging (mEGFP-dynein: 488 nm; mScarlet-NuMA^FL^: 561 nm) acquiring streams of 1,000 frames with 100 ms of exposure.

The imaging in Fig. 1, I and K was performed at 18°C. The imaging in Fig. 5, Fig. 6, and Video 2 was performed at 33°C. All other imaging was performed at 30°C. Imaging chamber heating was achieved using the OkoLab temperature control.

#### TIRF microscopy image processing

All acquired images were processed using Fiji (Schindelin et al., 2012) and FIESTA (Ruhnow et al., 2011). Drift correction and channel alignment were performed by tracking the position of TetraSpeck microspheres of 0.2 μm using FIESTA.

### Quantifications

#### Dynein activation

Dynein motility was analyzed by generating kymographs of the mEGFP-dynein signal for all the microtubules in every stream acquisition using FIESTA. Traces were manually drawn along all detectable straight diagonal lines in a kymograph. For dynein particles that alternated between processive motility and immobile states within the same run without detachment, the entire run was counted as representing an individual processive event. Medians and error bars shown in Fig. 1, D and G were obtained via bootstrapping using a custom MATLAB script. Briefly, events were counted for microtubules selected randomly among all biological replicates of the same condition, until the total measured microtubule length exceeded 100 µm; the mean number of events/100 µm/100 s was computed over 1,000 iterations. In each bootstrap iteration, a hyperbolic curve f(x)=ax/(b+x) was fitted to the bootstrap results at each condition (total 1,000 fitting results). Dynein velocities were automatically derived by the slope of each processive part in every trace.

#### Dynein processivity

For run length estimation, microtubule positions were determined using filament tracking in FIESTA. The tracked centerlines were corrected for color offset using atleast three reference TetraSpeck microspheres. Processive events along at least 10 microtubules were measured using the Kymograph Evaluation tool in FIESTA (Ruhnow et al., 2011). The empirical cumulative distribution function (CDF) was calculated in MATLAB using ecdf() and the survival probability (1-CDF) was fitted with an exponential function $f\left(x\right)=aexp^{b}$. The run length was calculated as R=−1/b and the error approximated by $∆R=2R/\sqrt{N}$.

To correct for processive events that reached the microtubule ends, the Kaplan–Meier estimator was used to calculate a corrected CDF using ecdf() and treating end events, i.e.,: processive events that end within 500 nm of the tracked microtubule end, as right-censored data points (Ruhnow et al., 2017). No correction for bleached events was applied.

#### NuMA fluorescence intensity on GMPCPP-microtubules

To quantify the fluorescence intensities of microtubule-bound mScarlet NuMA constructs, in the absence (Fig. 2, C and D) and presence of tubulin (Fig. 2, F and G), regions surrounding the microtubules were manually identified. The fluorescence intensity of all pixels within these regions was then measured in the mScarlet channel. Following background subtraction, the average fluorescence intensity per pixel was computed for each microtubule region, representing a single data point.

#### NuMA clustering propensity

To determine the amount of mScarlet NuMA clusters nonspecifically adsorbed to the glass surface during TIRF assays, three areas of equal dimensions were drawn within microtubule-free regions of each replicate image shown in Fig. 2 C. Using MATLAB, the fluorescence intensities of all pixels was measured in the mScarlet channel for each area. From the resulting intensity distribution (number of pixels at each different intensity value found in the image), outliers were detected through the generalized extreme studentized deviate (GESD) test (Rosner, 1983). Outlier intensities were background corrected (i.e., the median intensity was subtracted) and summed. The background corrected sum of all outlier intensities for one area corresponds to a single data point.

#### NuMA end selectivity on GMPCPP-microtubules

To assess the tendency of different mScarlet-NuMA constructs to bind the GMPCPP-microtubule end rather than the microtubule lattice in the presence of tubulin (Fig. 2, C and D), the mScarlet-NuMA and Atto647N-tubulin channels were overlaid. Microtubules showing mScarlet signal exclusively at one end were manually counted, and their percentage out of all microtubules with any mScarlet signal in one replicate image was calculated and represented a single data point.

#### Microtubule growth velocity

For each experimental condition shown in Fig. 3, Fig. 4, and Fig. S4, kymographs were created using FIESTA. The polymerization velocities were obtained by manually drawing a line along the growing ends and the velocity was automatically calculated from the slope of the line. Minus and plus ends were discerned from one another due to their significant velocity difference, with plus ends always growing faster than the minus ends. In case the ratio between the velocity of the two ends approximated 1, the microtubule was discarded from the analysis, likely being an antiparallel microtubule pair. When the end velocity was not consistent throughout the movie, all different velocities for one end were taken into account. The various segments of growth of a single microtubule end (marked by pauses or catastrophes/rescues) were considered to have different velocities simply when their slopes on a kymograph appeared clearly different from one another as judged by eye. This concerned almost exclusively the growth of minus ends in the presence of NuMA when a regularly growing end would slow down and/or stop upon NuMA landing and accumulation.

#### NuMA on γTuRC versus solution-nucleated microtubules

The binding frequency of NuMA to the minus end of γTuRC-nucleated and solution-nucleated microtubules (Fig. 5 C) was obtained by drawing kymographs of all microtubules in each experiment using FIESTA (line thickness: eight pixels) and manually counting the number of kymographs, where NuMA was observed to land on the minus end at or after nucleation, in the case of γTuRC-nucleated microtubules, and at or after microtubule landing, in the case of solution-nucleated microtubules. γTuRC-microtubules where NuMA was co-localized with γTuRC already before a detectable microtubule growth were not considered, as in control experiments in the presence of a low surface density of γTuRC (1 pM) no specific binding of NuMA to γTuRC was observed.

To quantify the maximum mScarlet fluorescence intensity at the minus end of γTuRC-nucleated and solution-nucleated microtubules (Fig. 5 D), for each minus end a surrounding area (8 × 8 pixels) was manually determined. To calculate the maximum mScarlet fluorescence intensity on the surface (Fig. 5 D), areas equal to those drawn around minus ends were identified at randomly chosen sites on the surface (20 per replicate) using a custom macro in Fiji; areas containing microtubules were discarded. For all areas, the maximum mScarlet intensity was then computed and background-corrected using the maximum intensity of an area of equal dimensions on the surface in the proximity of the minus end/random area of interest (not presenting any mScarlet nor microtubule signal). The frequency of non-specific NuMA landing onto microtubule-free areas on the surface (≈12%) was estimated by counting how many times the mScarlet signal was found within the above-mentioned randomly selected surface areas. A NuMA binding event was considered a landing event when NuMA appeared on the surface only after the beginning of the imaging and remained stably attached for the rest of the imaging.

For each nucleation assay shown in Fig. 5, E and F, microtubules were manually counted at six different time points using the “Multi-point tool” in Fiji. The total number of γTuRC-nucleated and solution-nucleated microtubules at a given time point was calculated by adding the newly nucleated microtubules to the total from the previous time point. To visualize single-molecule mBFP-γTuRC, the “Z project” function in Fiji was used to generate an average projection of the mBFP channel, which then served as a static background merged with the other channels.

#### NuMA capping of minus ends of γTuRC-released microtubules

A γTuRC-nucleated microtubule was considered to be treadmilling when, following γTuRC release triggered by KIF2A and spastin, it displayed minus-end depolymerization and a dynamic plus end, such that the microtubule seemed to translocate along the glass surface. γTuRC release frequency was measured by manually counting the percentage of treadmilling microtubules out of all γTuRC-nucleated microtubules.

The frequency of NuMA capping of γTuRC-released microtubules was calculated by manually counting the percentage of γTuRC-released microtubules for which treadmilling was followed by accumulation of NuMA at their minus end and consequential arrest of minus-end depolymerization. For such microtubules, NuMA mScarlet fluorescent signal was typically characterized by high intensity, similar to the intensity observed for NuMA capping the minus ends in GMPCPP-microtubules assays (Figs. 3 and 4).

Treadmilling and NuMA-capped microtubule lifetimes were calculated by measuring the time interval between γTuRC release and either microtubule disappearance due to complete depolymerization or the end of the movie (20 min).

#### NuMA binding to laser-ablated GMPCPP-microtubule ends

To assess the propensity of mScarlet-NuMA^FL^ to bind microtubule ends generated by laser ablation (Fig. 7 D), the mScarlet-NuMA and Atto647N-tubulin channels were overlaid. Only the microtubule segments that remained visible on the glass surface after the laser cut (≈37% of total cuts) were included in the analysis. The number of ablation-generated minus and plus ends displaying mScarlet-NuMA signal was manually counted. The abundance of each type of ablated end was expressed as a percentage of the total number of ablation-generated ends.

#### Dynein-driven lollipop microtubule transport

Landing events were manually counted and the frequency of each type of landing was expressed as a percentage of the total number of lollipops landed throughout the duration of the imaging (Fig. 8 E). For moving lollipops, being either anchored to the surface-immobilized microtubule only via their minus end (“single end contact”) or multiple contact points (“aligned parallel, “aligned antiparallel”), a transport event was defined as “processive” if, by drawing a kymograph in the microtubule channel: (1) a straight diagonal line associated to the lollipop movement over the immobilized microtubule could be detected, regardless of its length and slope; (2) the line co-localized in the dynein and NuMA channels. The lollipop minus end was identified by the only or most abundant NuMA signal along the lollipop; when identification was not possible, the transport was not counted. The minus end of immobilized microtubules was recognized by the minus-end directed processive motility of dynein and its resulting minus end accumulation. Dynein motility of non-lollipop attached dynein was likely activated by some NuMA that detached from lollipops and became available in solution.

A lollipop was considered as “stuck” if, since its appearance on the immobilized microtubule, its position did not change throughout the whole duration of the movie. Lollipops were labeled as “diffusing” when, upon landing, they wiggled back and forth along the immobilized microtubules, occasionally floating back in solution.

### Statistical tests

For measurements where each replicate of each condition corresponded to a single data point (Fig. 2 H, Fig. 6 D, and Fig. 7 D), the mean of all replicates was calculated and the means of different conditions were compared. For measurements where each replicate of each condition consisted of a relatively large number of data points (Fig. 1, E and H; Fig. 2, D, E, and G; Fig. 3, D, F, and H; Fig. 4, D, F, and H; Fig. 5 D; and Fig. S4 B), we considered only the median value for each replicate and compared the means of medians across different conditions. We assumed Gaussian distribution of residuals and we did not assume equal standard deviations.

To execute comparisons between more than two unpaired conditions (Fig. 1, E and H; Fig. 2 H; Fig. 3, D, F, and H; and Fig. 5 D), we selected Welch’s ANOVA test, with Holm-Sidak’s post-hoc test for multiple comparisons. We referred to the P values as “adjusted” when the values were corrected to take into account the overall risk of type I errors that come with multiple comparisons using the Dunnett T3 test. For single comparisons of unpaired data (Fig. 6 E and Fig. S4 B) and paired data (Fig. 4), we used Welch’s *t* test and paired *t* test, respectively.

All statistical tests were done using Prism 8 (GraphPad software). Population sizes refer to the sum of the *n* values of all replicates for each distinct condition.

### Data plots

Plots displayed in Fig. 1, D and G were obtained with MATLAB using a standard hyperbolic curve fit. Plots shown in Fig. 1, E and H were produced with the Violin SuperPlot package for MATLAB (Kenny and Schoen, 2021). Plots in Fig. S2, C and D were produced in MATLAB as previously described (Ruhnow et al., 2017). For all other figures, data plotting was performed using Prism 8 (GraphPad software).

### Online supplemental material

Fig. S1 shows the purified proteins used in dynein motility assays. Fig. S2 shows the effect of Lis1, temperature, and adaptor identity on dynein motility. Fig. S3 shows purified mScarlet-labeled NuMA C-terminal truncations. Fig. S4 shows the effect of NuMA^C-term S1^ at a high concentration on microtubule dynamics. Fig. S5 shows different modes of dynein/dynactin/NuMA^FL^-mediated microtubule transport. Video 1 shows that NuMA caps and stabilizes dynamic microtubule minus ends. Video 2 shows that NuMA gradually accumulates at the minus ends of enzymatically γTuRC-uncapped microtubule minus ends. Video 3 shows that NuMA binds to the minus ends of laser-ablated microtubules. Video 4 shows that full-length NuMA can mediate dynein/dynactin-driven microtubule transport.

## Supplementary Material

SourceData F2 is the source file for Fig. 2.

SourceData FS1 is the source file for Fig. S1.

SourceData FS3 is the source file for Fig. S3.

## Data Availability

The data are available from the corresponding author upon reasonable request.
