# Supplementary figures and images for: NuMA is a mitotic adaptor protein that activates dynein and connects it to microtubule minus ends
Source: J Cell Biol. 2025 Feb 11;224(4):e202408118. doi: 10.1083/jcb.202408118 (PMC11812572; doi:10.1083/jcb.202408118)

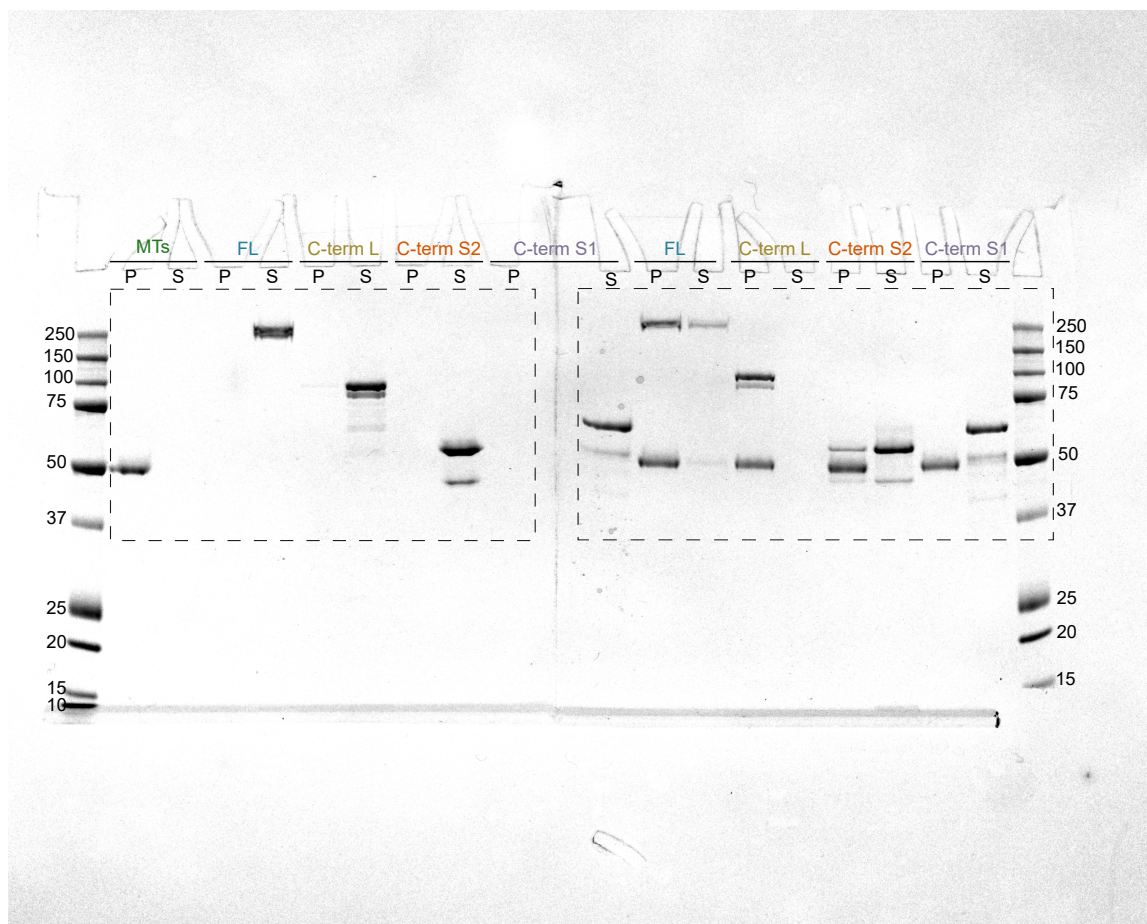

Supplement: SourceData F2 — is the source file for Fig. 2. [file jcb_202408118_sourcedataf2.pdf]

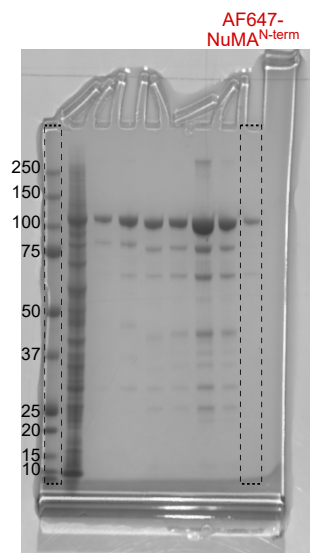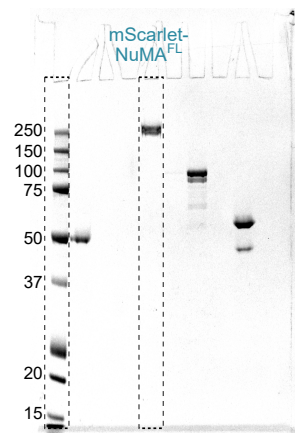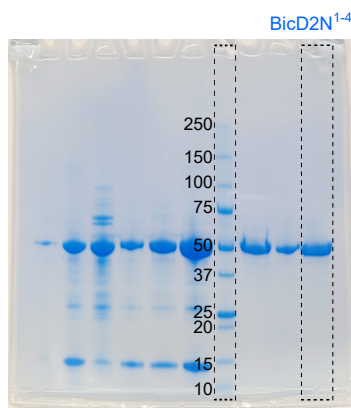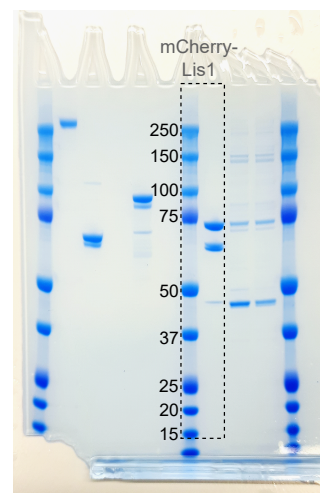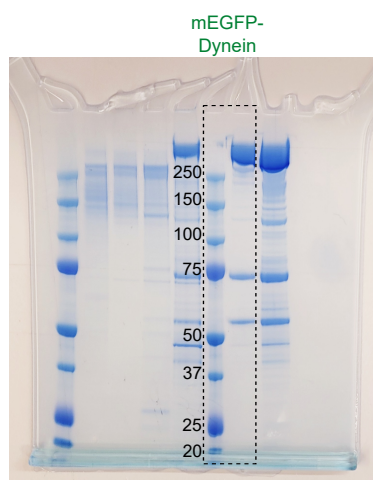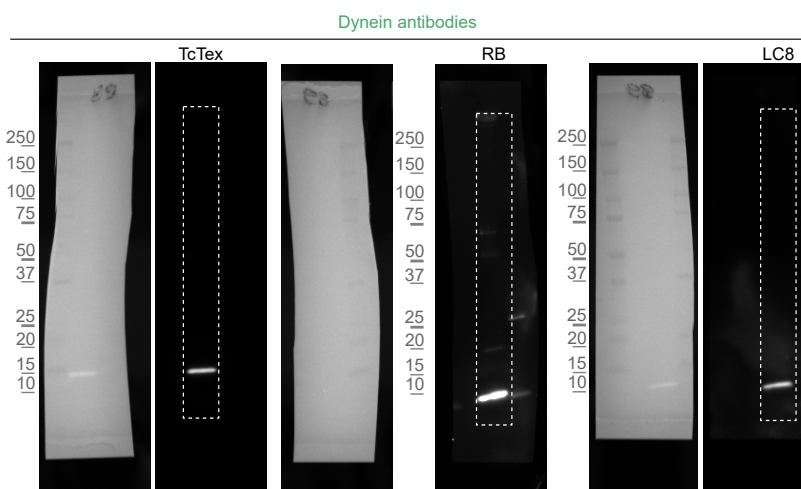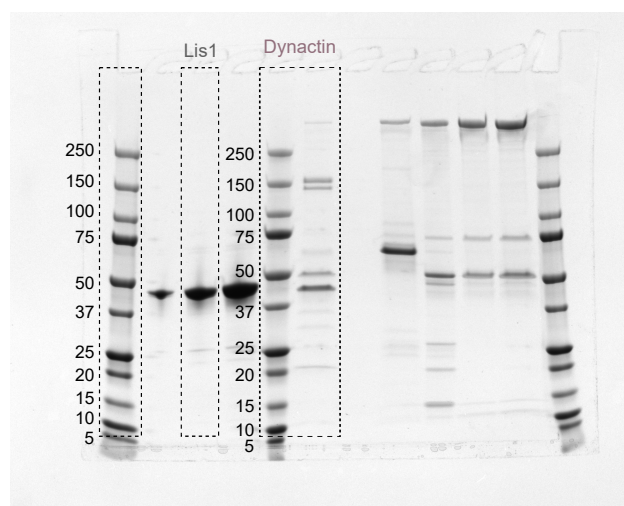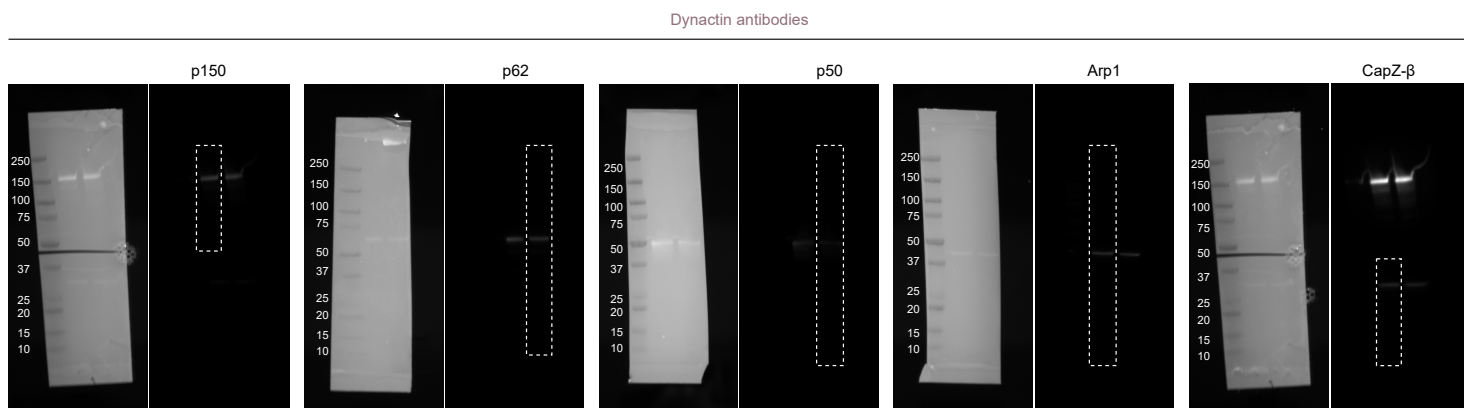

Supplement: SourceData FS1 — is the source file for Fig. S1. [file jcb_202408118_sourcedatafs1.pdf]
