# Supplementary material for: NuMA is a mitotic adaptor protein that activates dynein and connects it to microtubule minus ends
Source: J Cell Biol. 2025 Feb 11;224(4):e202408118. doi: 10.1083/jcb.202408118 (PMC11812572; doi:10.1083/jcb.202408118)
Supplement: SourceData FS3 — is the source file for Fig. S3. [file jcb_202408118_sourcedatafs3.pdf]

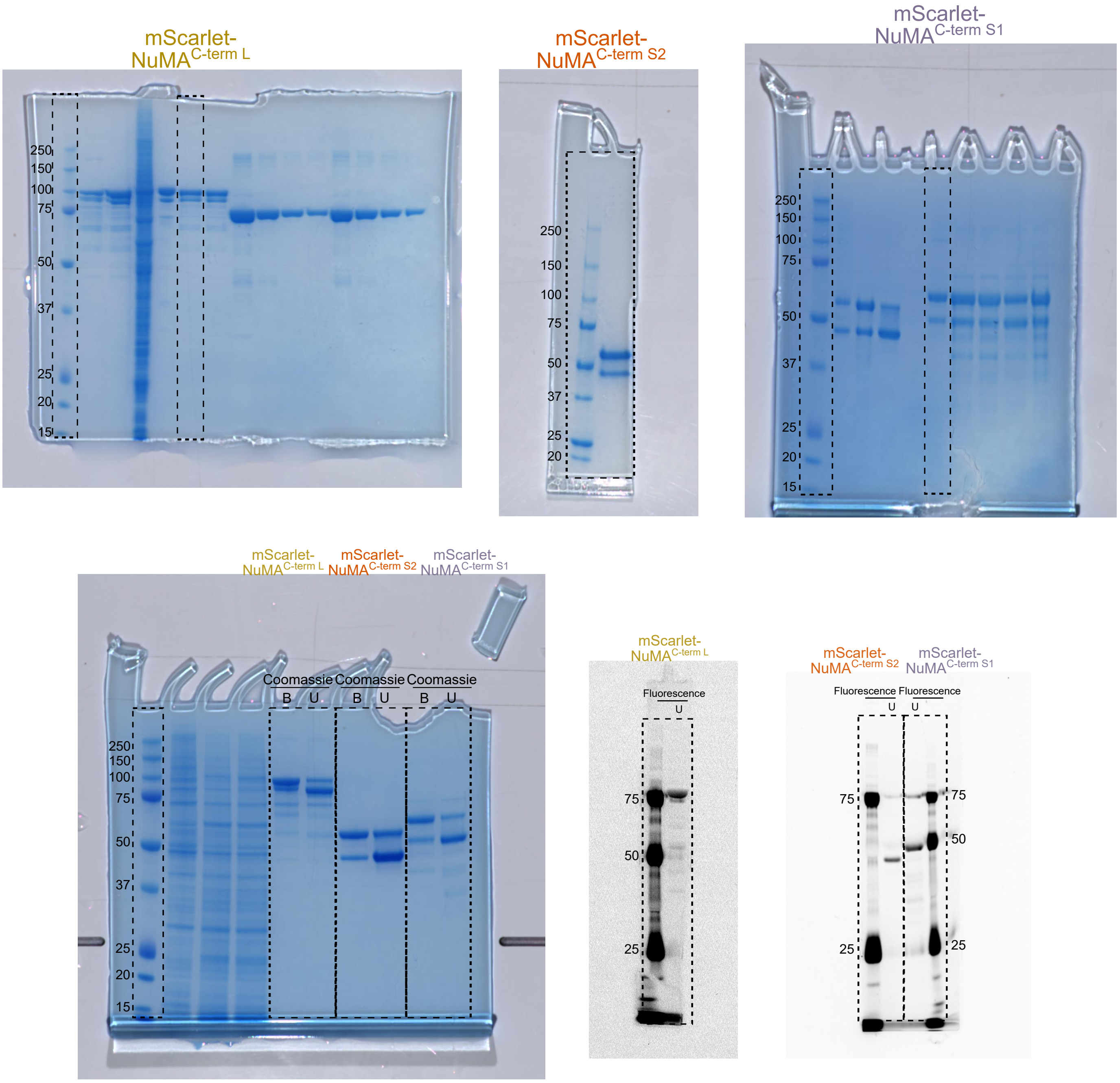
mScarlet-
NuMA C-term L
250
150
100
75
50
37
25
20
15
mScarlet-
NuMA C-term S2
250
150
100
75
50
37
25
20
mScarlet-
NuMA C-term S1
250
150
100
75
50
37
25
20
15
mScarlet-
NuMA C-term L
mScarlet-
NuMA C-term S2
mScarlet-
NuMA C-term S1
Coomassie Coomassie Coomassie
B U B U B U
250
150
100
75
50
37
25
20
15
mScarlet-
NuMA C-term L
Fluorescence
U
75
50
25
mScarlet-
NuMA C-term S2
mScarlet-
NuMA C-term S1
Fluorescence Fluorescence
U U
75
50
25
